# Phenylketonuria from the perspectives of patients in Türkiye

**DOI:** 10.1186/s13023-024-03079-z

**Published:** 2024-02-20

**Authors:** Merve Esgi, Hakan Ergun, Nazmi Yalcin Kaya, Deniz Yilmaz Atakay, Ege Erucar, Fatma Celik

**Affiliations:** 1 Department of Nutrition and Dietetics, Faculty of Health Sciences, Kocaeli University, Kocaeli, Türkiye; 2 Department of Medical Pharmacology, Ankara University Faculty of Medicine, Ankara, Türkiye; 3 Omega-HelCon, Ankara, Türkiye; 4 PKU Family Association, Istanbul, Türkiye; 5 Faculty of Medicine, Altinbas University, Istanbul, Türkiye; 6 Food and Drug Department, Parma University, Parma, Italy

**Keywords:** Phenylketonuria, Phenylalanine-restricted diet, Medical nutrition products, Unmet needs, New treatments

## Abstract

**Background:**

The present study aimed to determine the problems, unmet needs and expectations of phenylketonuria (PKU) patients in Türkiye regarding follow-up and treatment in order to provide data for future planning and implementations on PKU.

**Methods:**

The study included patients diagnosed with PKU and/or their parents. They were informed about the study via phone calls and their verbal consents were obtained. Questions in the data collection forms, which were established separately for pediatric, adolescent, and adult age groups, were applied during the interviews and the answers were recorded.

**Results:**

Among 182 classical PKU patients, 66 (36.3%) were in the pediatric group (0–12 years old), 44 (24.2%) were in the adolescent group (13–19 years old), and 72 (39.5%) were in the adult group (≥ 20 years old). In all patient groups, phenylalanine-restricted diet and medical nutrition products were the main options for treatment. The median of the last measured blood phenylalanine concentration (patient-reported) was 290 µmol/L, 425 µmol/L, and 750 µmol/L in the pediatric, adolescent, and adult groups, respectively. The frequency of blood testing for serum phenylalanine level according to the age groups was appropriate in nearly half of the patients. While the majority of the patients have been visiting the metabolism center they have been diagnosed with PKU for control, considerable proportion of the patients would like to change the center or the doctor they visit for control if they could. It was determined that nearly half of the patients had trouble in accessing the metabolism center. Treatment options’ being limited and expensive were the major problems. The main requests of the patients and patient relatives included easier access to the metabolism centers and more options for treatment and diet.

**Conclusions:**

Access to the services should be easier to improve the patients’ follow-up and treatment. There is need for low-cost, easily applicable, and accessible nutrition products and effective novel pharmacological agents. Focusing on these issues in health policies by providing pedagogic/psychological support, establishing support programs also comprising the families, and increasing the awareness activities were the key outcomes.

**Supplementary Information:**

The online version contains supplementary material available at 10.1186/s13023-024-03079-z.

## Background

Phenylketonuria (PKU) is an autosomal recessive metabolic disease. Despite the variations across the world, the prevalence of PKU is approximately 1/10.000 newborns [1]. In patients with PKU, phenylalanine in the protein-containing foods cannot be degraded because of deficiency or lack of phenylalanine hydroxylase enzyme leading to phenylalanine accumulation in the blood and in the tissues, which results in brain damage in particular. It causes various neuropsychiatric problems such as severe mental failure, epilepsy and behavioral disorders unless treated [1]. Newborn screening is essential for early diagnosis. Phenylalanine-restricted diet is the fundamental basis of PKU treatment; in addition, there are pharmacologic therapeutic options (such as synthetic tetrahydrobiopterin and pegylated ammonium lyase). Other options including gene therapy and mRNA therapies are under development [1, 2]. In order to achieve optimal clinical and neuropsychological outcomes, serum phenylalanine levels should be kept under control for lifetime. The burden caused by the treatment of a disease starting just after birth and lasting for life has an impact on social life and quality of life of both patients and their families [1].

Türkiye is one of the countries where PKU is the most prevalent. The data from the Ministry of Health have revealed that one of each 4.500 neonates is born with PKU and it is estimated that approximately 200 new babies with PKU join the community each year [3]. However, scientific researches revealing the needs of patients and patient relatives regarding PKU are lacking in Türkiye. The present study aimed to identify the patients’ problems and unmet needs about treatment and follow-up, as well as to inquire their expectations to provide data for future planning and implementations concerning PKU. It is estimated that an insight at national level will be provided about patient journey from birth to adulthood with the data to be obtained.

## Results

A total of 182 classical PKU patients were enrolled in the study. Three different age groups were established as follows: (I) Pediatric group: newborns/infants/children aged 0–12 years; (II) Adolescent group: adolescents aged 13–19 years; (III) Adult group: adults aged ≥ 20 years. Of the patients, 66 (36.3%) were in the pediatric group, 44 (24.2%) were in the adolescent group, and 72 (39.5%) were in the adult group. General characteristics of the patient groups are demonstrated in Table 1.

**Table 1:** General characteristics of PKU patients

	Patient Group					
		Pediatric		Adolescent		Adult
	**N**	Median (Q1-Q3) (Min.-Max.)	**N**	Median (Q1-Q3) (Min.-Max.)	**N**	Median (Q1-Q3) (Min.-Max.)
Age, year,	66	8 (6–10) (2–12)	44	14 (13–17) (13–18)	72	23 (22–26) (20–47)
Sex	66	n (%)	44	n (%)	72	n (%)
Female		34 (51.5)		22 (50.0)		39 (54.2)
Male		32 (48.5)		22 (50.0)		33 (45.8)
Mean household monthly income, €	65	n (%)	41	n (%)	60	n (%)
< 312.5		23 (35.4)		12 (29.3)		17 (28.3)
312.5–625		33 (50.8)		20 (48.8)		27 (45.0)
> 625–1250		6 (9.2)		8 (19.5)		14 (23.3)
> 1250		3 (4.6)		1 (2.4)		2 (3.3)
Patient’s caregiver *	66	n (%)	44	n (%)	72	n (%)
Mother		65 (98.5)		42 (95.5)		47 (65.3)
Father		13 (19.7)		14 (31.8)		11 (15.3)
Sibling		2 (3.0)		3 (6.8)		6 (8.3)
None (i.e. self-care)		-		1 (2.3)		34 (47.2)
Other (grandmother/spouse)		5 (7.6)		1 (2.35)		5 (6.9)

*more than one option is available

Information on the frequency of follow-up and the service received by the patients at the metabolism centers is summarized in Table 2. As expected, the follow-up visits were more frequent in the pediatric age group. The main service received at the metabolism centers was measurement of phenylalanine concentration. It was observed that the proportion of patients receiving pedagogue/psychologist support was higher in the adolescent group. The median (Q1-Q3) of the last measured blood phenylalanine concentration (patient-reported) was 290 (209–420) µmol/L in the pediatric group, 425 (275–800) µmol/L in the adolescent group, and 750 (545–910) µmol/L in the adult group. The last measured phenylalanine level was higher than the target level in adult patients.

**Table 2:** Information on PKU patients’ follow-up

		Pediatric		Adolescent		Adult
	**N**	n (%)	**N**	n (%)	**N**	n (%)
**Frequency of measuring blood phenylalanine concentration**	66		43		71	
Once in a year		-		2 (4.7)		20 (28.2)
Every six months		5 (7.6)		6 (14.0)		6 (8.5)
Every three months		12 (18.2)		7 (16.3)		10 (14.1)
Once in a month		15 (22.7)		20 (46.5)		24 (33.8)
Twice in a month		32 (48.5)		6 (14.0)		4 (5.6)
Other		2 (3.0)		2 (4.7)		7 (9.9)
**Number of follow-ups at metabolism center in the last year**	55	n (%)	31	n (%)	43	n (%)
1		27 (49.1)		12 (38.7)		32 (74.4)
2		19 (34.5)		15 (48.4)		6 (14.0)
3		5 (9.1)		2 (6.5)		3 (7.0)
≥ 4		4 (7.3)		2 (6.5)		2 (4.7)
Service received at the metabolism center *	66	n (%)	44	n (%)	72	n (%)
Measurement of serum phenylalanine concentration		64 (97.0)		41 (93.2)		70 (97.2)
Recommendations for low-protein diet and diet calculation		47 (71.2)		31 (70.5)		61 (84.7)
Dose adjustment for medical therapy		40 (60.6)		27 (61.4)		51 (70.8)
Other laboratory tests		34 (51.5)		30 (68.2)		59 (81.9)
Support from Pedagogue/Psychologist		2 (3.0)		7 (15.9)		6 (8.3)
		n (%)		n (%)		n (%)
**Regular visits for dietician control**	66	59 (89.4)	44	33 (75.0)	72	56 (77.8)
**Receiving support from Pedagogue/Psychologist**	66	15 (22.7)	44	29 (65.9)	72	27 (37.5)
		Mean ± SD Median (min-max)		Mean ± SD Median (min-max)		Mean ± SD Median (min-max)
The last measured serum phenylalanine level, µmol/L**	33	347.12 ± 194.75 290 (100–840)	27	603.26 ± 558.52 425 (110–3000)	49	745.00 ± 315.00 750 (90-1694)

* more than one option is available, ** this data was obtained by questioning the patient/families

The problems that the patients experience at the metabolism centers, and their satisfaction from the center and specialists providing service are presented in Table 3. In general, the patients seemed to have high satisfaction with the services received from metabolism centres. Nevertheless, it was determined that they had difficulties in accessing the services.

**Table 3:** The problems experienced at the metabolism centers and the level of patient satisfaction

		Pediatric		Adolescent		Adult
	**N**	n (%)	**N**	n (%)	**N**	n (%)
**Those who are still visiting the same center where they have been diagnosed with PKU**	66	61 (92.4)	44	31 (70.5)	72	65 (90.3)
**Those who would like to change the center or the specialist they visit for control if they can**	66	32 (48.5)	43	19 (44.2)	71	21 (29.6)
**Those who have trouble in accessing the metabolism center**	66	39 (59.1)	44	25 (56.8)	72	24 (33.3)
Reasons for trouble in accessing the metabolism center*	66		44		72	
It is difficult to make an appointment		27 (40.9)		16 (36.4)		16 (22.2)
Very long waiting time for examination /control		24 (36.4)		15 (34.1)		20 (27.8)
Metabolism center is located in another city		21 (31.8)		10 (22.7)		4 (5.6)
Economic hardship		21 (31.8)		16 (36.4)		10 (13.9)
Difficulty in accommodation because the center is located in another city		15 (22.7)		5 (11.4)		2 (2.8)
Other		4 (6.1)		5 (11.4)		6 (8.3)
**Level of satisfaction from the metabolism center providing service**	66		44		71	
Very satisfied		8 (12.1)		10 (22.7)		19 (26.8)
Satisfied		36 (54.5)		19 (43.2)		44 (62.0)
Dissatisfied		22 (33.3)		15 (34.1)		8 (11.3)
**Level of satisfaction from the metabolism specialist providing service**	66		44		71	
Very satisfied		10 (15.2)		8 (18.2)		21 (29.6)
Satisfied		45 (68.2)		27 (61.4)		47 (66.2)
Dissatisfied		11 (16.7)		9 (20.5)		3 (4.2)

*more than one option is available

Treatments performed in the patients to keep the phenylalanine concentration under control and related difficulties are summarized in Table 4. Majority of the patients were using phenylalanine-restricted diet and medical nutrition products. However, most patients reported that the products were expensive, difficult to apply, and limited in options. It was determined that sapropterin response testing was performed in only 12 patients and sapropterin treatment was applied in only 3 patients.

**Table 4:** Information on treatment of the patients

		Pediatric		Adolescent		Adult
	**N**	n (%)	**N**	n (%)	**N**	n (%)
Treatment for phenylalanine control *	66		44		72	
None		1 (1.5)		2 (4.5)		2 (2.8)
Phenylalanine-restricted diet		56 (84.8)		37 (84.1)		61 (84.7)
Medical nutrition products		41 (62.1)		33 (75.0)		50 (69.4)
Large neutral amino acid (LNAA)		4 (6.1)		1 (2.3)		12 (16.7)
Sapropterin		-		1 (2.3)		2 (2.8)
Difficulties in implementing phenylalanine-restricted diet *	66		44		72	
None		5 (7.6)		2 (4.5)		4 (5.6)
The products are expensive		57 (86.4)		41 (93.2)		54 (75.0)
Options are limited		43 (65.2)		27 (61.4)		49 (68.1)
Difficulty in implementation at school		37 (56.1)		21 (47.7)		30 (41.7)
Difficulties in implementation due to work life /education/trip, etc.		34 (51.5)		24 (54.5)		40 (55.6)
Lack of taste/flavor		30 (45.5)		17 (38.6)		29 (40.3)
It is difficult to prepare/no recipe		24 (36.4)		16 (36.4)		27 (37.5)
Low/limited efficacy in lowering phenylalanine levels		22 (33.3)		10 (22.7)		23 (31.9)
Reaction of the people around because of diet type		23 (34.8)		13 (29.5)		26 (36.1)
Difficulties in using medical nutrition products *	65		41		72	
None		8 (12.3)		3 (7.3)		12 (16.7)
Products are expensive		47 (72.3)		37 (90.2)		53 (73.6)
Limited options		33 (50.8)		27 (65.9)		42 (58.3)
Difficulties in implementation due to work life /education/trip, etc.		24 (36.9)		19 (46.3)		37 (51.4)
Lack of taste/flavor		31 (47.7)		20 (48.8)		28 (38.9)
Low/limited efficacy in lowering phenylalanine levels		19 (29.2)		15 (36.6)		16 (22.2)
Other		5 (7.7)		3 (7.3)		3 (4.2)
Difficulties in using amino acid or LNAA *	66		44		72	
None		45 (68.2)		32 (72.7)		58 (80.6)
Poor taste		19 (28.8)		11 (25)		9 (12.5)
Low/limited efficacy in lowering phenylalanine levels		5 (7.6)		5 (11.4)		2 (2.8)
Difficulty in using due to high number of tablets		5 (7.6)		4 (9.1)		2 (2.8)
**Those who underwent Sapropterin response testing**	63	5 (7.9)	39	2 (5.1)	68	5 (7.4)

* more than one option is available

The unfavorable situations/symptoms that the patients have experienced are summarized in Table 5. The most common emotions experienced by the patients were found as angry/irritable mood and failure to concentrate/difficulty in focusing. Approximately half of the patients stated that there were things they wanted but could not do because of illness.

**Table 5:** Unfavorable situations/symptoms that patients have experienced

		Pediatric		Adolescent		Adult
	**N**	n (%)	**N**	n (%)	**N**	n (%)
Unfavorable situations/symptoms that the patient have experienced *	66		44		72	
None		21 (31.8)		4 (9.1)		13 (18.1)
Angry/irritable mood		27 (40.9)		26 (59.1)		38 (52.8)
Sad mood		14 (21.2)		13 (29.5)		24 (33.3)
Rebellious mood		12 (18.2)		9 (20.5)		14 (19.4)
Anxious mood		11 (16.7)		9 (20.5)		20 (27.8)
Indecisive mood		10 (15.2)		9 (20.5)		23 (31.9)
Laziness or feeling of laziness		11 (16.7)		14 (31.8)		25 (34.7)
Feeling of tiredness		7 (10.6)		15 (34.1)		30 (41.7)
Failure to concentrate / difficulty in focusing		18 (27.3)		22 (50.0)		27 (37.5)
Difficulty in understanding the subjects in class/at work/daily life		12 (18.2)		18 (40.9)		20 (27.8)
Difficulty in communicating with others		8 (12.1)		11 (25.0)		12 (16.7)
Slow reaction		4 (6.1)		6 (13.6)		8 (11.1)
Feeling like in a smokescreen		2 (3.0)		5 (11.4)		8 (11.1)
Headache		4 (6.1)		8 (18.2)		16 (22.2)
Vision impairment		4 (6.1)		6 (13.6)		11 (15.3)
Gastric complaints		4 (6.1)		5 (11.4)		12 (16.7)
Other		3 (4.5)		6 (13.6)		3 (4.2)
**Presence of comorbidity according to Charlson Comorbidity index**	66	1 (1.5)	44	4 (9.1)	72	8 (11.1)
**Those who stated that there are things they want but could not do because of illness**	66	34 (51.5)	44	23 (52.3)	71	37 (52.1)
**Those who consider their illness as an obstacle for social life**	66	28 (42.4)	44	14 (31.8)	71	20 (28.2)

* more than one option is available

The topics that are lacking for or desired to have more information or being informed about concerning PKU disease are demonstrated in Table 6. The topics that the patients mentioned most were new treatment and diet options.

**Table 6:** The topics lacking for and/or desired to have more information about

		Pediatric		Adolescent		Adult
	**N**	n (%)	**N**	n (%)	**N**	n (%)
Novel therapies	66	50 (75.8)	44	32 (72.7)	72	50 (69.4)
New dietary opportunities (such as recipes)	66	46 (69.7)	44	31 (70.5)	72	51 (70.8)
More frequent contact with doctor	66	40 (60.6)	44	35 (79.5)	72	35 (48.6)
Food contents	66	39 (59.1)	44	29 (65.9)	72	43 (59.7)
Campaigns for disease awareness	66	38 (57.6)	44	25 (56.8)	72	31 (43.1)
Group activities for patients and patient relatives	66	37 (56.1)	44	26 (59.1)	72	39 (54.2)
Patient societies	66	35 (53.0)	44	26 (59.1)	72	29 (40.3)
Other	66	6 (9.1)	44	3 (6.8)	72	4 (5.6)

Information on the facilities that the patients want to have is presented in Table 7. It was observed that patients’ expectations were high in almost every aspect of their illness, namely treatment options, follow-up opportunities (easy access to the center, easy blood sampling), and being informed.

**Table 7:** Facilities the patients want to have

		Pediatric		Adolescent		Adult
	**N**	n (%)	**N**	n (%)	**N**	n (%)
Activation of more metabolism centers for ease of access	66	60 (90.9)	44	40 (90.9)	72	52 (72.2)
More options for low-protein foods	66	59 (89.4)	44	41 (93.2)	72	60 (83.3)
Programs about PKU and newborn screening on television/TV series	66	59 (89.4)	44	41 (93.2)	72	62 (86.1)
Raising awareness about phenylketonuria in the society	66	58 (87.9)	44	41 (93.2)	72	56 (77.8)
Opportunity of sending blood samples to metabolism center for phenylalanine measurement	66	58 (87.9)	44	36 (81.8)	72	54 (75.0)
Availability of home devices for phenylalanine measurement	66	57 (86.4)	44	40 (90.9)	72	56 (77.8)
Availability of more options for drug therapy	66	57 (86.4)	44	39 (88.6)	72	51 (70.8)
Opportunity of control/follow-up without visiting a hospital (control via phone-call and internet /follow-up system, blood collection at home, etc.)	66	55 (83.3)	44	40 (90.9)	72	57 (79.2)
Awareness activities in every province	66	55 (83.3)	44	39 (88.6)	72	53 (73.6)
Providing the patient families with educational opportunity on PKU management	66	51 (77.3)	44	33 (75.0)	72	46 (63.9)
More active patient society	66	51 (77.3)	44	34 (77.3)	72	45 (62.5)
Other	66	2 (3.0)	44	2 (4.5)	72	4 (5.6)

## Discussion

It has been mentioned at the PKU State-of-the-Science Conference in 2012 that newborn screening for PKU has a history of more than 50 years and there is an aging population of adult PKU patients. In addition, it was also stated that there are many questions yet to be clarified about diagnosis, treatment and long-term outcomes in PKU patients and further studies on this subject are needed [4]. Based on the fact that studies investigating the patient needs are limited in number in Türkiye, one of the countries where PKU is the most prevalent, we aimed to identify the PKU patients’ problems and needs experienced in the diagnosis and treatment during childhood, adolescence, and adulthood.

There is yet no curative treatment for PKU, and the patients have to follow a lifelong diet program. Regular monitoring of blood phenylalanine concentration and keeping it at target level with diet and/or medical therapy are essential and of critical importance. It has been demonstrated that starting the treatment at an early stage and continuing efficiently in older ages improves neuropsychological and cognitive outcomes [5–9]. Good adherence to treatment keeps blood phenylalanine concentration under control, reduces complications, and improves executive functions [10, 11]. There is a consensus on keeping the phenylalanine concentration at 120–360 µmol/L since birth in pediatric PKU patients. Nevertheless, US guidelines recommend the blood phenylalanine concentrations to be kept at 120–360 µmol/L in the patients aged > 12 years (excluding pregnant women), whereas European guidelines recommends keeping it at 120–600 µmol/L [12, 13]. In the present study, the median (Q1-Q3) of the last measured blood phenylalanine concentration (patient-reported) was 290 µmol/L (209–420 µmol/L) in the pediatric group, 425 µmol/L (275–800 µmol/L) in the adolescent group, and 750 mol/L (545–910 µmol/L) in the adult group. It has been reported that adherence to treatment among PKU patients is better in the first years of life (probably due to maternal control of feeding) but decreases close to the adolescent period [7, 10]. It has been also claimed that PKU patients are largely lost to follow-up at the metabolism centers as they become adults [14]. According to the results of the present study, blood phenylalanine concentration increases with age; thus, it can be concluded that adherence to diet and/or phenylalanine control decreases with age. Results of a survey study comprising 24 countries highlighted the need for further researches and recommendations/guidelines on therapeutic target for the management of adult PKU patients and pointed out the need for identification of the patients lost to follow-up for lifelong proper management of the disease [15].

Considering the services received at the metabolism centers, it was determined that “measurement of blood phenylalanine concentration” is the service most commonly received in all of the three groups. Besides, nutrition and diet recommendations, dose adjustment for medical therapy, and other laboratory tests are also among the services received at the centers. Since the diet plays an important role in the management of PKU, it is essential for dieticians/nutritionist and for the physicians to be involved at this point [16, 17]. In the present study, it was found that most of the patients (89.4% in the pediatric group, 75% in the adolescent group, and 77.8% in the adult group) regularly visit a dietician for control.

Although patient monitoring programs varies among countries, frequency of measuring the blood phenylalanine concentration recommended in the literature is once a week for 0-2-year-old infants, once a week or every two weeks for 3-9-year-old toddlers, every two weeks or every month for 10-15-year-old adolescents, and every month or every two months for ≥ 16-year-old adults. Clinical evaluation is suggested to be performed every 1–3 months in 0-2-year-old infants, every 3–6 months in 3-9-year-old toddlers, every six months in 10-15-year-old adolescents, and every year in ≥ 16-year-old adults [18]. Although different from the age groups mentioned above [18], considering the age groups in the present study, the frequency of blood phenylalanine measurement appears to be in line with the recommendations in nearly half of the patients. The number of follow-ups at the metabolism center in the last year was under the recommended frequencies particularly in the pediatric group. Given that the study participants are the patients who are still in contact with the patient society, it can be concluded that the frequency of follow-up may be lower across the country or in other patients who are not in contact with the patient society. It has been reported that individuals who are likely to discontinue treatment can be identified early and measures that would provide continuity of treatment can be taken in order to enhance the patients’ adherence to treatment and monitoring programs [19].

It is seen that the great majority of patients (92.4% in the pediatric group, 70.5% in the adolescent group and 90.3% in the adult group) are still visiting the metabolism center where they have been diagnosed with PKU for control. Most of the patients (66.6% in the pediatric group, 65.9% in the adolescent group, and 88.8% in the adult group) declared that they are satisfied/very satisfied with the center or specialist they receive service from. Nevertheless, it can be stated that considerable amount of patients would like to change the center or the specialist they visit for control if they could (48.5% in the pediatric group, 44.2% in the adolescent group, and 29.6% in the adult group). It was determined that nearly half of the patients had difficulty in accessing the metabolism centers and that the center’ being located in another city, economic hardship, difficulty in making appointment, and long waiting period were the major reasons declared. Although satisfaction rates with services is high, patients and families still have expectations about improvements on several topics. This can be due to the facts that PKU is a rare disease with few treatment options and that PKU is an ongoing disease since birth and requires special care, which negatively affects the psychology of the families, which results in high expectations.

Evaluating the treatment status of PKU patients, phenylalanine-restricted diet and medical nutrition products were identified as the primary options. With regard to the problems experienced with phenylalanine-restricted diet and medical nutrition products, the most frequently mentioned problem in all patient groups was the inaccessibility of products due to high cost. In addition, limited options and difficulty in implementation were also declared as important problems. It has been reported that patient quality of life is improved when patient adherence is high with large neutral amino acid (LNAA) therapy, which is one of other treatment options [20]. In the present study, significant proportion of patients stated that they have no difficulty in using LNAA therapy; however, the number of patients using this therapy remains very low. Sapropterin is also another option for pharmacologic treatment of PKU [21]. In the present study, according to the participants’ statement, sapropterin response testing has been performed in 7.9% of the patients in the pediatric group, 5.1% of the patients in the adolescent group, and 7.4% of the patients in the adult group. Again, the rate of sapropterin use was low in the patient groups included in this study. More effective, user friendly, and easily accessible pharmacological agents, and new medical foods are needed for the treatment of PKU. With the introduction of such agents, it may be easier to take the disease under control [22].

It has been reported that mood alterations, such as anxiety and depression, are more prevalent in PKU patients, particularly in those with higher phenylalanine concentrations than recommended [10, 23]. In the present study, angry/irritable mood was the most frequent unfavorable symptom reported in all age groups, followed in the order of frequency by failure to concentrate/difficulty in focusing and sad mood in pediatric group; failure to concentrate/difficulty in focusing and difficulty in understanding the subjects in the class/at work/daily life in the adolescent group; feeling of tiredness and failure to concentrate/difficulty in focusing in the adult group. It has been also reported that PKU patients have difficulties in social relations [10]. In the present study, the question “have you (has he/she) ever had anything that you (he/she) would like to do but could not because of your (his/her) illness?” was answered “yes” by nearly half of the patients (about 52% in all age groups). The rate of the answer given as “yes” to the question “Is phenylketonuria a barrier for your social life?” was 42.4% in the pediatric age group, 31.8% in the adolescent group, and 28.2% in the adult group. In the present study, the ratio of patients receiving pedagogic/psychological support was 22.7% in the pediatric group, 65.9% in the adolescent group, and 37.5% in the adult group. Based on these results, the adolescent patients were observed to receive more pedagogic/psychological support. Self-advocacy begins in adolescence. In our country, school time occupies a large portion of adolescents’ time. Moreover, during the adolescence period, children encounter a social environment outside the family and school and goes to restaurants and cafes as a means of socialization, where the children may feel themselves a social misfit, the reasons of which can be not being able to find suitable food on the menu or having to explain the diet to their friends. Accordingly, compliance of the adolescent patients to their diets may become difficult both in school and in social environments and thereby causing them to have problems in expressing themselves. As the result, adolescents may require more pedagogic/psychological support in order to cope with the disease. It is reported that PKU patients and their families are in need of long-term psychological support while trying to overcome this chronic problem and that hospital staff should not be left alone in meeting this need and support programs that help to develop strategies to cope with daily problems are necessary [24].

The prevalence of comorbidity is higher in PKU patients as compared to the general population, and evaluation in terms of comorbidity should be a part of disease management particularly in adult patients [25]. In the present study, the prevalence of comorbidity was 1.5% in the pediatric group, 9.1% in the adolescent group, and 11.1% in the adult group.

It is known that PKU disease affects not only the quality of life of the patients but also affects the quality of life of their families’ [26–28]. Having support is also important for the families to cope with the problems in the management of PKU. When the patients and patients’ relatives were questioned about the issues they feel lacking or want to have more information about, the following issues were most commonly stated in all groups; novel therapies, new nutritional opportunities, and having the opportunity of more frequent contact with the doctor. Regarding the opportunities that the patients and patients’ relatives would like to have, it was determined that they most commonly want activation of more metabolism centers for ease of access. In addition, availability of more options for treatment and diet, transportation facilities for sending blood to the center for phenylalanine measurement or availability of measurement at home, possibility of control/follow-up without visiting a hospital, providing the families with the opportunity of education on PKU management, and more active patient society were among the requests of majority of the patients. This shows us that the patients’ feelings about the issues they feel lacking are extremely consistent with their demands.

In the present study, the fact that the majority of study participants are the members of PKU Family Association from the Marmara Region can be considered a limitation. This requires caution while generalizing the results for the whole country. Nevertheless, it can be concluded that unmet needs may be at the same level, even higher, across the country and/or for the patients who are not in contact with the patient society. We believe that the results of the present study would be a very critical source of data for future studies. Data obtained from this study can be used by researchers to conduct more focused studies on patients’ follow-up, support, care, treatment, and social life. We concluded that focusing particularly on the issues which the patients and patient relatives would like to get information about, establishing solutions for these issues, and assessing the fields to be developed within the frame of health policy and service delivery would make favorable contributions to disease management.

## Conclusions

PKU is a disease that requires life-long monitoring. According to the results of the present study, the frequency of patient follow-up is not sufficient, and more and/or easily accessible metabolism centers/specialists are needed and in-place patient follow-up methods should be provided to overcome this problem. Although satisfaction rates with health services is high, patients and families still have expectations about improvements on several topics. In order to improve adherence to treatment, there is a need for increasing the number low-cost, easily applicable and accessible options of medical nutrition products and more efficient novel pharmacological agents. Patients and patients’ relatives should be informed about current diagnostic and therapeutic opportunities in collaboration with patient society, specialist society, and the centers. Pedagogic/psychological support programs should be developed and generalized. Patients should be monitored closely also for comorbidities, particularly as they are getting old. Strategies that would also support the families should be developed to improve PKU management and to enhance patient quality of life. Population-wide PKU awareness activities should be increased.

## Methods

### Patients

This study was conducted between September 22, 2020 and October 23, 2020. The study was approved by the Human Research Ethics Committee of Ankara University Faculty of Medicine (approval date: 17.06.2020, approval no: İ5-314-20). After obtaining the ethics committee approval, PKU patients who signed the informed consent form were included. The patients were accessed through the registry of PKU Family Association. The list provided by the PKU Family Association consisted of 554 patient records. Of these records, 464 were from the Marmara region. In order to provide a balance in geographical distribution, 113 patients were included from the Marmara region and 69 patients were included from the other regions of Türkiye; thus, a total of 182 patients were reached. Information on infants and pediatric patients was obtained from their parents.

### Procedures

The patients/parents were informed about the study by phone calls by the researcher. Study informed consent form and data collection form were posted to the subjects who considered to participate in the study. It was planned that the forms would be sent back again by post after being completed. However, it was realized that some patients or patients’ relatives refrained from receiving/opening and/or sending back the forms due to concerns about infection because of COVID-19 pandemic during the study period. Accordingly, the research protocol was changed and the Ethics Committee approval was revised. The patients were called by phone, informed consent forms were read and their verbal consents were obtained. Questions in the data collection form were asked during the phone calls and the answers were recorded. The personnel conducting the phone calls received a standard education prior to the study. The phone calls conducted using Computer-Assisted Telephone Interviewing (CATI) system were recorded and securely stored.

### Forms

All questions obtained from the PKU patient society and from similar studies were pooled, and three different data collection forms were structured for three different age groups (pediatric, adolescent and adult) in accordance with study objectives. The pediatric, adolescent, and adult data collection forms were provided in the Additional pdf file 1, Additional pdf file 2, and Additional pdf file 3, respectively, both in Turkish and English.

Charlson comorbidity index [29] was used to determine the prevalence of comorbidities.

### Statistical analysis

Data analysis was performed using the PASW Statistics 18.0 (SPSS Inc., Chicago, IL, USA) for Windows program. Descriptive statistics were expressed as number and percentage for categorical variables and as mean, standard deviation, median, minimum, maximum, percentile 25 (Q1), and percentile 75 (Q3) for numerical variables.

### Electronic supplementary material

Below is the link to the electronic supplementary material.

**Additional file 1**: The pediatric data collection form in Turkish and English.

**Additional file 2**: The adolescent data collection form in Turkish and English.

**Additional file 3**: The adult data collection form in Turkish and English.

## Data Availability

The data that support the findings of this study are available from the corresponding author, [ME], upon reasonable request.

## Abbreviations

PKU: Phenylketonuria
CATI: Computer-Assisted Telephone Interviewing
