# Supplementary material for: Phenylketonuria from the perspectives of patients in Türkiye
Source: Orphanet J Rare Dis. 2024 Feb 20;19:78. doi: 10.1186/s13023-024-03079-z (PMC10880278; doi:10.1186/s13023-024-03079-z)
Supplement: Supplementary file 1 — Additional file 1: The pediatric data collection form in Turkish and English. [file 13023_2024_3079_MOESM1_ESM.pdf]

Fenilketonüri (PKU) Hasta Yolculuğu Araştırması

Veri Toplama Formu

Çocukluk Çağı (0-12 Yaş)

ANKETÖR (isminizin yanına çarpı işareti koyunuz)

S.D. ☐

G.Ç. ☐

Hasta Ad/Soyadı Baş Harfleri ....

Ebeveynin Adı/Soyadı Baş Harfleri ....

Anket doldurma Tarihi ve Saat

....

AÇIKLAMALAR

Klasik Fenilketonüri tanısı almış ve hakkında bilgi toplanan kişi "***Çocuğunuz***", çocuğunuzun bu soru formuna yanıt veren siz ebeveynleri ise "***Yakını***" olarak tanımlanmaktadır.

GÖNÜLLÜ SEÇİM ÖLÇÜTLERİ

ÇALIŞMAYA DAHİL ETME ÖLÇÜTLERİ

	Evet	Hayır
1. Klasik Fenilketonüri tanısı almış (0-12 yaş arası) çocuklar	☐	☐
2. Çalışma hakkında bilgilendirilen ve yazılı olur veren hasta yakını	☐	☐
3. Araştırma kapsamında kullanılacak veri toplama formunda bulunan sorulara ve değerlendirmelere yanıt vermek için entelektüel kapasiteye sahip olan hasta yakını	☐	☐

Çocuğun çalışmaya alınabilmesi için yukarıdaki soruların tümünün cevabı **E V E T** olmalıdır!

ÇALIŞMA DIŞI BIRAKMA ÖLÇÜTLERİ

	Evet	Hayır
1. Çalışmaya katılmayı istemeyenler.	☐	☐

Çocuğun çalışmaya alınabilmesi için yukarıdaki soruların tümünün cevabı **H A Y I R** olmalıdır!

I. ÇOCUĞUNUZA AİT TANIMLAYICI BİLGİLER

1. Doğum Tarihi	.... / .... / ....<br>Gün Ay Yıl	
2. Cinsiyeti	☐ Erkek	☐ Kız
3. Yaşadığı İl	……………………..……………..……………….……	
4. Okuduğu Okul	……………………..……………..……………….……	
5. Çocuğunuzun sağlığı ile evde yaşayanlardan kim ilgileniyor	☐ Anne<br>☐ Kardeş<br>☐ Baba<br>☐ Diğer (açıklayınız): ………….……………..…………..…………	
6. Hane halkının aylık ortalama geliri	☐ Asgari Ücret seviyesi (2.324 TL)<br>☐ 2.500 - 5.000 TL arası<br>☐ 5.001 - 10.000 TL arası<br>☐ 10.001 – 15.000 TL arası<br>☐ 15.001 TL üstü	

**II. METABOLİZMA MERKEZİNE AİT BİLGİLER**	
7. Sürekli olarak kontrole götürdüğünüz Metabolizma Merkezinin adı nedir?	☐ İstanbul Üniversitesi Tıp Fakültesi - İstanbul<br>☐ Cerrahpaşa Tıp Fakultesi - İstanbul<br>☐ Okmeydanı Eğitim ve Araştırma Hastanesi - İstanbul<br>☐ Marmara Üniversitesi Tıp Fakültesi - İstanbul<br>☐ Hacettepe Üniversitesi Tıp Fakültesi - Ankara<br>☐ Ankara Üniversitesi Tıp Fakültesi - Ankara<br>☐.Gazi Üniversitesi Tıp Fakültesi - Ankara<br>☐.Başkent Üniversitesi Çocuk Hastanesi - Ankara<br>☐.Ankara Şehir Hastanesi - Ankara<br>☐ Dokuz Eylül Üniversitesi Tıp Fakültesi - İzmir<br>☐ Ege Üniversitesi Tıp Fakültesi - İzmir<br>☐ Akdeniz Üniversitesi Tıp Fakültesi - Antalya<br>☐ Antalya Eğitim ve Araştırma Hastanesi - Antalya<br>☐ Çukurova Üniversitesi Tıp Fakültesi - Adana<br>☐ Adana Şehir Hastanesi - Adana<br>☐ Uludağ Üniversitesi Tıp Fakültesi - Bursa<br>☐ Van Eğitim Araştırma hastanesi - Van<br>☐ Diğer ……….………………….……<br><br>☐ **HİÇ BİR** Metabolizma Merkezine kontrole götürmüyoruz gitmiyoruz.<br>(*8. soruya geçiniz*)
8. Çocuğunuzu “son iki yılda” kontrol için kaç kez Metabolizma Merkezine götürdünüz?	☐ 1 ☐ 2<br>☐ 3 ☐ 4<br>☐ Daha fazla (belirtiniz): ……….
9. Çocuğunuzu “son bir yılda” kontrol için kaç kez Metabolizma Merkezine götürdünüz?	☐ 1 ☐ 2<br>☐ 3 ☐ 4<br>☐ Daha fazla (*belirtiniz*): ……….………….…………..
10. Halen Fenilketonüri tanısı konulan Metabolizma Merkezine mi kontrole gidiyorsunuz?	☐ **EVET**, hâlâ aynı Metabolizma Merkezine kontrole gidiyoruz *(11. soruya geçiniz)*<br>☐ **HAYIR** farklı bir Metabolizma Merkezine kontrole gidiyoruz *(12. soruya geçiniz)*<br>☐ **HİÇ BİR** Metabolizma Merkezine kontrole götürmüyoruz *(13. soruya geçiniz)*
11. Aynı Metabolizma Merkezine ne sıklıkla gidiyorsunuz?	☐ Son üç yıldır **her yıl İKİ KEREDEN FAZLA** sayıda kontrole gidiyoruz<br>☐ Son üç yıldır **her yıl EN AZ BİR KERE** kontrole gidiyoruz<br>☐ Diğer (*açıklayınız*): ………..………..………….. (*14. soruya geçiniz*)
12. Başka bir Metabolizma Merkezine ne sıklıkla gidiyorsunuz?	☐ Son üç yıldır **her yıl İKİ KEREDEN FAZLA** sayıda kontrole gidiyoruz<br>☐ Son üç yıldır **her yıl EN AZ BİR KERE** kontrole gidiyoruz<br>☐ Diğer (*açıklayınız*): ………..………..………….. (*14. soruya geçiniz*)

13. Metabolizma Merkezine neden gitmiyorsunuz?	☐ İhtiyaç duymadık<br>☐ Gitmemiz gerektiğini kimse söylemedi<br>☐ İmkanımız yok<br>☐ Diğer (*açıklayınız*): ………….………….…………..<br>(*14. soruya geçiniz*)
14. İmkanınız olsa kontrole gittiğiniz Metabolizma Merkezi ya da Metabolizma Uzmanını değiştirmek ister miydiniz?	☐ Evet, değiştirmek isterdim.<br>(*15. soruya geçiniz*)
	☐ Hayır, değiştirmek istemezdim.<br>(*16. soruya geçiniz*)
15. Nedenini belirtiniz	…….…………………..……….…….……………….……
16. Düzenli olarak **<u>diyetisyen kontrolüne</u>** gidiyor musunuz?	☐ Evet düzenli olarak gidiyoruz (*17. soruya geçiniz*)
	☐ Hayır düzenli olarak gitmiyoruz (*18. soruya geçiniz*)
	☐ Hiçbir diyetisyene kontrole gitmiyoruz. (*18. soruya geçiniz*)
17. Diyetisyene ne sıklıkla kontrole gidiyorsunuz?	☐ **Her yıl İKİ KEREDEN FAZLA** sayıda kontrole gidiyoruz<br>☐ **Her yıl EN AZ BİR KERE** kontrole gidiyoruz<br>☐ Diğer (*açıklayınız*): ………….………….…………..
18. Diyetisyene neden gitmiyorsunuz?	☐ İhtiyaç duymadık<br>☐ Gitmemiz gerektiğini kimse söylemedi<br>☐ Diyeti kendi isteğimizle bıraktık<br>☐ Diyeti bırakmamız söylendi<br>☐ Tedavi bırakıldı<br>☐ İmkanımız yok<br>☐ Gerekli olmadığını düşünüyoruz<br>☐ Diğer (*açıklayınız*): ………….………….…………..
19. **<u>Pedagog</u>** desteği aldınız mı?	☐ **Evet** Pedagoga gittik<br>(*20. soruya geçiniz*)
	☐ **Hayır** Pedagoga gitmedik<br>(*21. soruya geçiniz*)
20. Pedagoga ne sıklıkla gidiyorsunuz?	☐ **Her yıl İKİ KEREDEN FAZLA**<br>☐ **Her yıl EN AZ BİR KERE**<br>☐ Diğer (*açıklayınız*): ………….………….…………..
21. Pedagoga neden gitmediniz?	☐ İhtiyaç duymadık<br>☐ Gitmemiz gerektiğini kimse söylemedi<br>☐ İmkanımız yok<br>☐ Gerekli olmadığını düşünüyoruz<br>☐ Diğer (*açıklayınız*): ………….………….…………..

22. Şu anda hizmet aldığınız Metabolizma Merkezinden memnuniyet durumunuz nedir?	☐ Çok memnunuz<br>☐ Memnunuz<br>☐ Memnun değiliz (*Nedenini belirtiniz*):<br>…………..…………..…..………..…………..…..………<br>…………..…………..…..………..…………..…..………
23. Şu anda hizmet aldığınız Metabolizma Uzmanından memnuniyet durumunuz nedir?	☐ Çok memnunuz<br>☐ Memnunuz<br>☐ Memnun değiliz (*Nedenini belirtiniz*):<br>…………..…………..…..………..…………..…..………<br>…………..…………..…..………..…………..…..………
24. Metabolizma Merkezinden aldığınız hizmetler nelerdir?	☐ Fenilalanin kan seviyesi ölçümü<br>☐ Diğer laboratuvar testleri<br>☐ Tıbbi tedavi doz ayarlaması<br>☐ Düşük proteinli Beslenme ve diyet hesaplaması önerileri<br>☐ Pedagog desteği<br>☐ Diğer (*açıklayınız*): ………..………..…………..
25. Eğer veriliyor olsaydı Metabolizma Merkezinden almak istediğiniz diğer hizmetler neler olurdu?	Açıklayınız:<br>…………..…………..…..………..…………..…..………<br>…………..…………..…..………..…………..…..………<br>…………..…………..…..………..…………..…..………<br>…………..…………..…..………..…………..…..………
26. Fenilalanin kontrolü için kullandığınız seçenekler hangileridir?<br><br>(*sizin için uygun birden fazla seçenek var ise işaretleyebilirsiniz.*)	☐ Fenilalanin kısıtlı diyet<br>☐ Tıbbi amaçlı diyet ürünleri<br>☐ Büyük Nötral Aminoasit (LNAA)<br>☐ Sapropterin<br>☐ Diğer (*açıklayınız*): ………..………..…………...
27. Fenilalanin kısıtlı diyet uygulamasında karşılaştığınız zorluklar nelerdir?<br><br>(*sizin için uygun birden fazla seçenek var ise işaretleyebilirsiniz.*)	☐ Seçeneklerin az olması<br>☐ Hazırlamanın zor olması / tarif bilgilerinin olmaması<br>☐ Fenilalanin seviyesini düşürmede etkisinin az/kısıtlı olması<br>☐ Beslenme şekli sebebiyle çevreden tepki alması<br>☐ Beslenme şeklinin okulda zorluklara sebep olması<br>☐ Tad ve lezzet eksikliği<br>☐ Ürünlerin pahalı olması nedeniyle erişilememesi<br>☐ Çalışma hayatı, eğitim, seyahat gibi nedenlerle diyete uyumun sosyal zorlukları (*Yaşadığınız sosyal zorluğu belirtiniz*)<br>…………..…………..…..………..…………..…..………<br>…………..…………..…..………..…………..…..………<br>…………..…………..…..………..…………..…..………<br>…………..…………..…..………..…………..…..………<br>☐ Diğer (*açıklayınız*):<br>…………..…………..…..………..…………..…..………<br>…………..…………..…..………..…………..…..………<br>…………..…………..…..………..…………..…..………<br>…………..…………..…..………..…………..…..………<br>☐ Hayır, yok

28. Tıbbi amaçlı diyet ürünlerinin kullanımında karşılaştığınız zorluklar nelerdir?<br><br>(sizin için uygun birden fazla seçenek var ise işaretleyebilirsiniz.)	☐ Seçeneklerin az olması<br>☐ Fenilalanin seviyesini düşürmede etkisinin az/kısıtlı olması<br>☐ Tad ve lezzet eksikliği<br>☐ Pahalı olması<br>☐ Çalışma hayatı, eğitim, seyahat gibi nedenlerle diyete uyumun sosyal zorlukları (*Yaşadığınız sosyal zorluğu belirtiniz*)<br>……………………………………………………………<br>……………………………………………………………<br>……………………………………………………………<br>……………………………………………………………<br>☐ Diğer (*açıklayınız*): …………………………………...
29. Amino Asit veya LNAA kullanımında karşılaştığınız zorluklar nelerdir?<br><br>(sizin için uygun birden fazla seçenek var ise işaretleyebilirsiniz.)	☐ Tatlarının kötü olması<br>☐ Fenilalanin seviyesini düşürmede etkisinin az/kısıtlı olması<br>☐ Tablet sayısının fazla olması nedeniyle kullanım güçlüğü<br>☐ Diğer (*açıklayınız*): …………………………………
30. Çocuğunuza PKU tedavisi için Sapropterin duyarlılık testi yapıldı mı?	☐ Hayır (*31. soruya geçiniz*)<br>☐ Evet (*32. soruya geçiniz*)
31. Yapılmamasının nedenini belirtiniz:	……………………………………………………………<br>……………………………………………………………<br>……………………………………………………………<br>(*34. soruya geçiniz.*)
32. Sapropterin duyarlılık testi pozitif çıktıysa size bu ilaç reçete edilerek kullanmanız sağlandı mı?	☐ Evet<br>☐ Hayır<br>Nedenini belirtiniz: …………………………………………<br>……………………………………………………………
33. Sapropterin tedavisi uygularken karşılaştığınız zorluklar var mı, varsa nelerdir?<br><br>(sizin için uygun birden fazla seçenek var ise işaretleyebilirsiniz.)	☐ Gönüllünün Sapropterin duyarlı olmaması<br>☐ Fenilalanin seviyesini düşürmede etkisinin az/kısıtlı olması<br>☐ Yan etkileri<br>☐ Diğer (*açıklayınız*): …………………………………<br>☐ YOK
34. Çocuğunuzu kontrol için farklı bölüm uzmanlarına götürüyor musunuz?<br><br>Cevabınız EVET ise hangi bölüme - yılda kaç kez gittiğinizi yazar mısınız?	☐ Evet (*35. soruya geçiniz*)<br>☐ Hayır (*36. soruya geçiniz*)
35. Çocuğunuzu kontrol için hangi uzmanlara, yılda kaç kez götürüyorsunuz?	☐ Psikiyatrist: ………………….kez/yıl<br>☐ Nörolog: ……………………kez/yıl<br>☐ Diğer (*açıklayınız*): …………………………………
36. Metabolizma merkezine erişimde zorluk yaşıyor musunuz?	☐ Evet (*37. soruya geçiniz*)<br>☐ Hayır (*38. soruya geçiniz*)

37. Metabolizma merkezine erişimde ne tür zorluklar yaşıyorsunuz?<br><br>(sizin için uygun birden fazla seçenek var ise işaretleyebilirsiniz.)	☐ Metabolizma merkezinin başka şehirde olması<br>☐ Merkezin başka şehirde olması nedeniyle test sonuçları ve tedavi doz ayarlaması için ertesi gün konaklama yapma zorunluluğu olması<br>☐ Randevu almanın zorlukları olması<br>☐ Muayene ve kontrol için bekleme süresinin çok uzun olması<br>☐ Ekonomik zorluklar olması<br>☐ Diğer (*açıklayınız*): ………….…………..…………..….	
38. Çocuğunuzun kan fenilalanin değeri kontrolünü hangi kurum aracılığı ile sağlıyorsunuz	☐ Metabolizma merkezi<br>☐ Devlet hastanesi<br>☐ Özel hastane<br>☐ Özel laboratuvar<br>☐ Diğer (*açıklayınız*): ………….…………..…………..….	
39. Çocuğunuzun kan fenilalanin değerini ne sıklıkla takip ediyorsunuz?	☐ Yılda bir<br>☐ 6 ayda bir<br>☐ 3 ayda bir	☐ Ayda bir<br>☐ Ayda iki<br>☐ Diğer (*açıklayınız*):<br>…………..…………………
40. Doktorunuzun ya da diyetisyenin, çocuğunuzun, beyin gücünü kullanabilmesi için belirlediği hedef fenilalanin düzeyi nedir?	☐ 240 µmol/L<br>☐ 360 µmol/L<br>☐ 480 µmol/L	☐ 600 µmol/L<br>☐ Diğer (*açıklayınız*):<br>…………..…………………
41. Çocuğunuzun kan fenilalanin ölçümü en son kaç ay önce yapıldı?	☐ 1 ay önce<br>☐ 2 ay önce	☐ 3 ay önce<br>☐ Diğer (*açıklayınız*):<br>…………..…………………
42. Çocuğunuzun en son ölçülen kan fenilalanin değeri nedir?	Belirtiniz: ……… µmol/L	
43. Hasta yakınının eksikliğini hissettiği, daha çok bilgi sahibi olmayı da bilgilendirilmeyi istediği konular nelerdir?<br><br>(sizin için uygun birden fazla seçenek var ise işaretleyebilirsiniz.)	☐ Hastalık farkındalığı kampanyaları<br>☐ Hasta dernekleri<br>☐ Yeni tedaviler<br>☐ Yeni beslenme olanakları (yemek tarifleri gibi)<br>☐ Gıda içerikleri<br>☐ Hasta ve yakınlarına ait grup aktiviteleri<br>☐ Doktor ile daha sık görüşmek<br>☐ Diğer (*açıklayınız*): ………….…………..…………..….	

44. Hasta yakınının sahip olmak istediği **DİĞER İMKANLAR** nelerdir?<br><br>(sizin için uygun birden fazla seçenek var ise işaretleyebilirsiniz.)	☐ Hasta ailelerine PKU yönetimi konusunda eğitim olanaklarının sağlanması<br>☐ Erişim kolaylığı için daha fazla Metabolizma Merkezinin faaliyete geçmesi<br>☐ Metabolizma merkezine fenilalanin ölçümü için kan gönderme imkanı olması<br>☐ Hastaneye gitmeden telefon ve internet ile takip ve kontrol sistemi kurulması (evde kan alma, uzaktan fenilalanin ve uzman kontrolü)<br>☐ Hasta Derneğinin daha aktif olması<br>☐ Her ilde farkındalık faaliyetlerinin yapılması<br>☐ Fenilalanin seviyesinin ölçülmesi için Evde ölçüm cihazlarının bulunması<br>☐ Daha fazla ilaç tedavisi seçeneği olması<br>☐ Daha fazla düşük proteinli gıda seçeneği olması<br>☐ Toplumda fenilketonüri konusunda farkındalık yaratılması<br>☐ TV de, dizilerde PKU, yenidoğan taraması anlatılması.<br>☐ Diğer (*açıklayınız*): …………..…………..……………..…

**III. SOSYAL YAŞAMA AİT ÖZELLİKLER**		
45. Çocuğunuzun **MEVCUT DURUMDA** yaşadığı olumlu / olumsuz bulgular var mı? Var ise nelerdir?<br><br>(sizin için uygun birden fazla seçenek var ise işaretleyebilirsiniz.)	☐ Ders dinlerken dikkat toplayamama / odaklanma güçlüğü<br>☐ Dersi anlamada güçlük çekme<br>☐ Öfkeli / sinirli ruh hali<br>☐ Endişeli ruh hali<br>☐ Üzüntülü ruh hali<br>☐ İsyankar ruh hali<br>☐ Tembellik ya da tembellik hissi<br>☐ Yorgunluk hissi<br>☐ Kararsız ruh hali<br>☐ Reaksiyon yavaşlığı<br>☐ Sis perdesi içinde olduğunu hissetmek<br>☐ Başkaları ile İletişim güçlüğü<br>☐ Baş ağrısı<br>☐ Görme bozukluğu<br>☐ Mide şikayetleri<br>☐ Diğer (*açıklayınız*): …………..…………..………….<br>☐ **YOK**	
46. Çocuğunuzun hastalığı sebebiyle yapmak isteyip yapamadığı bir şey oldu mu?	☐ Evet oldu (*47. soruya geçiniz.*)<br>☐ Hayır olmadı (*48. soruya geçiniz.*)	
47. Çocuğunuzun hastalığı sebebiyle yapmak isteyip yapamadığı şeyi veya şeyleri yazınız.	1) ……………………………………………………………<br>2) ……………………………………………………………<br>3) ……………………………………………………………<br>4) ……………………………………………………………<br>5) ……………………………………………………………	
48. Çocuğunuzun hastalığı sosyal yaşantısı için bir engel mi?	☐ Evet	☐ Hayır
49. Çocuğunuz ne kadar sıklıkla öfkeleniyor?	☐ Çok sık<br>☐ Sık	☐ Nadiren<br>☐ Hiç
50. Çocuğunuz öfkesini kontrol edememesi nedeniyle herhangi bir kötü olaya maruz kaldı mı?	☐ Evet	☐ Hayır
51. Çocuğunuz ne kadar sıklıkla kendisini mutsuz hissediyor?	☐ Çok sık<br>☐ Sık	☐ Nadiren<br>☐ Hiç
52. Çocuğunuz unutkanlık yaşıyor mu?	☐ Evet (*53. soruya geçiniz.*)<br>☐ Hayır (*54. soruya geçiniz.*)	
53. Ne sıklıkla unutkanlık yaşıyor?	☐ Çok sık<br>☐ Sık	☐ Nadiren
54. PKU hastası olmayan yaşayan başka çocuğunuz var mı?	☐ Evet (*55. ve 56. soruyu yanıtlayınız.*)<br>☐ Hayır (**IV. Eşlik Eden Kronik Hastalık Bilgileri** bölümüne geçiniz.)	

55. Sağlıklı çocuğunuzla PKU hastası çocuğunuz karşılaştırdığınızda yaşadığınız zorluklar nelerdir?	………………………………………………………………<br>………………………………………………………………<br>………………………………………………………………
56. Sağlıklı çocuğunuzla PKU hastası çocuğunuzun dersleri ve sınavlarındaki başarıları farklılık gösteriyor mu?	☐ EVET farklılık var. PKU hastası çocuğumun başarısı daha düşük<br>☐ HAYIR farklılık yok.<br>☐ EVET farklılık var. PKU hastası çocuğumun başarısı daha yüksek

**IV. EŞLİK EDEN KRONİK HASTALIK BİLGİLERİ**	
57. Çocuğunuzda bulunan ve yan tarafta listelenen hastalık ya da hastalıkları işaretleyiniz.<br><br>Çocuğunuzda birden fazla hastalığın olması halinde ilgili her hastalığın yanına işaret koyunuz.	☐ Miyokart enfarktüsü (geçirilmiş kalp krizi)<br>☐ Kalp yetmezliği<br>☐ Periferik damar hastalığı (varis, damar tıkanıklığı)<br>☐ Kalp-damar hastalığı<br>☐ Demans (bunama)<br>☐ Kronik akciğer hastalığı (astım, KOAH, tüberküloz)<br>☐ Romatizmal hastalık (romatizma)<br>☐ Peptik ülser (ülser)<br>☐ Karaciğer hastalığı<br>☐ Diyabet (şeker hastalığı)<br>☐ Hemipleji ya da parapleji (kısmı ya da tam felç)<br>☐ Böbrek hastalığı<br>☐ Malignite (kanser hastalığı)<br>☐ AIDS/HIV

Phenylketonuria: The Patient’s Journey Study

Data Collection Form

Pediatric Group (Between 0 and 12 Years of Age)

INTERVIEWER (Put a cross next to your name)

S.D. ☐

G.Ç. ☐

Initials of the Patient’s Name/Surname **Initials of the Parent's Name/Surname**

....

Survey Completion Date and Time

....

EXPLANATIONS

The person who has been diagnosed with Classical Phenylketonuria and about whom information is collected is defined as "***Your Child***", and you, the parents of your child who responded to this questionnaire, are defined as "***Relatives***".

VOLUNTEER SELECTION CRITERIA

STUDY INCLUSION CRITERIA

	Yes	No
1. Children diagnosed with Classical Phenylketonuria (0-12 years old)	☐	☐
2. The patient's relative who was informed about the study and gave written consent	☐	☐
3. The patient's relative who has the intellectual capacity to answer the questions and evaluations in the data collection form to be used within the scope of the study.	☐	☐

In order for the child to be included in the study, the answer to all of the above questions must be **Y E S!**

STUDY EXCLUSION CRITERIA

	Yes	No
1. Those who do not want to participate in the study.	☐	☐

In order for the child to be included in the study, the answer to all of the above questions must be **N O!**

I. DESCRIPTIVE INFORMATION ABOUT YOUR CHILD

1. Date of Birth	.... / .... / ....<br>Day Month Year	
2. Gender	☐ Boy	☐ Girl
3. City of residence	………………………………………………………………	
4. The school he/she attended	………………………………………………………………	
5. Among the people living at home, who takes care about your child's health?	☐ Mother<br>☐ Sibling<br>☐ Father<br>☐ Other (please explain): ……………………………………………	
6. Average monthly income of the household	☐ Minimum Wage level (2.324 TL)<br>☐ Between 2.500 - 5.000 TL<br>☐ Between 5.001 - 10.000 TL<br>☐ Between 10.001 – 15.000 TL<br>☐ Over 15.001 TL	

**II. INFORMATION ABOUT THE METABOLISM CENTER**	
7. What is the name of the Metabolism Center where you regularly take your child for control?	☐ İstanbul University School of Medicine - İstanbul<br>☐ Cerrahpaşa School of Medicine - İstanbul<br>☐ Okmeydanı Training and Research Hospital - İstanbul<br>☐ Marmara University School of Medicine - İstanbul<br>☐ Hacettepe University School of Medicine - Ankara<br>☐ Ankara University School of Medicine - Ankara<br>☐.Gazi University School of Medicine - Ankara<br>☐.Başkent University Children's Hospital - Ankara<br>☐.Ankara City Hospital - Ankara<br>☐ Dokuz Eylül University School of Medicine - İzmir<br>☐ Ege University School of Medicine - İzmir<br>☐ Akdeniz University School of Medicine - Antalya<br>☐ Antalya Training and Research Hospital - Antalya<br>☐ Çukurova University School of Medicine - Adana<br>☐ Adana City Hospital - Adana<br>☐ Uludağ University School of Medicine - Bursa<br>☐ Van Training and Research Hospital - Van<br>☐ Other ………..…………………..……<br><br>☐ **Neither of them** We do not go to a Metabolism Center for control.<br>(*Go to question 8*)
8. How many times have you taken your child to the Metabolism Center for control in the "last two years"?	☐ 1 ☐ 2<br>☐ 3 ☐ 4<br>☐ More (specify)………
9. How many times have you taken your child to the Metabolism Center for control in the "last year"?	☐ 1 ☐ 2<br>☐ 3 ☐ 4<br>☐ More (specify)………
10. Are you still visiting the same Metabolism Center that made the diagnosis of Phenylketonuria for control?	☐ **YES**, we still visit the same Metabolism Center for control. *(Go to question 11)*<br>☐ **NO,** we are visiting a different Metabolism Center for control. *(Go to question 12)*<br>☐ **Neither of them** We do not visit a Metabolism Center for control (*Go to question 13)*
11. How often do you visit the same Metabolism Center?	☐ We have been going for control **MORE THAN TWICE every year** for the last three years.<br>☐ We have been going for control **AT LEAST ONCE a year** for the last three years.<br>☐ Other (please explain): ……………………………….<br>*(Go to question 14)*

12. How often do you go to another Metabolism Center?	☐ We have been going for control **MORE THAN TWICE every year** for the last three years<br>☐ We have been going for control **AT LEAST ONCE a year** for the last three years.<br>☐ Other (please explain) ………...………...……….. ...<br>(*Go to question 14*)
13. Why don't you go to a Metabolism Center?	☐ We didn't need to.<br>☐ Nobody told us that we should go<br>☐ We don't have financial means<br>☐ Other (please explain) ………...………...……….. ...<br>(*Go to question 14*)
14. If you had the opportunity, would you want to change the Metabolism Center or Metabolism Specialist you visit for control?	☐ Yes, I would like to change.<br>(*Go to question 15*)
	☐ No, I wouldn't want to change.<br>(*Go to question 16*)
15. Please state the reason	………………………………………………………
16. Do you **visit a dietitian** regularly?	☐ Yes, we go regularly (*Go to question 17*)
	☐ No, we don't go regularly (*Go to question 18*)
	☐ We do not visit any dietician for control. (*Go to question 18*)
17. How often do you visit a dietitian for control?	☐ We are going for control **MORE THAN TWICE every year**<br>☐ We are going for control **AT LEAST ONCE a year**<br>☐ Other (please explain) ………...………...……….. ...
18. Why don't you visit a dietician?	☐ We didn't need to<br>☐ Nobody told us that we should go<br>☐ We left the diet voluntarily.<br>☐ We were told to stop dieting<br>☐ Treatment was stopped<br>☐ We don't have the financial means<br>☐ We think it is not necessary<br>☐ Other (please explain) ………...………...……….. ...
19. Have you received **pedagogical support** ?	☐ **Yes,** we visited a Pedagog**.**<br>(*20. Go to question*)
	☐ **No,** we didn't visit a Pedagog<br>(*Go to question 21*)
20. How often do you visit a Pedagog?	☐ **MORE THAN TWICE every year**<br>☐ **AT LEAST ONCE a year**<br>☐ Other (please explain) ………...………...……….. ...

21. Why didn't you visit a Pedagog?	☐ We didn't need to<br>☐ Nobody told us that we should go<br>☐ We don't have the financial means<br>☐ We think it is not necessary<br>☐ Other (please explain) …………...…………...……………...
22. What is your satisfaction level with the Metabolism Center you are currently receiving service from?	☐ We are very satisfied<br>☐ We are satisfied<br>☐ We are not satisfied (specify why)...………………….……………….……………….…………<br>…………...…………...…...………….………….…...………
23. What is your satisfaction level with the Metabolism Specialist you are currently receiving service from?	☐ We are very satisfied<br>☐ We are satisfied<br>☐ We are not satisfied (specify why):<br>…………...…………...…...………….………….…...………<br>…………...…………...…...………….………….…...………
24. What services do you receive from the Metabolism Center?	☐ Phenylalanine blood level measurement<br>☐ Other laboratory tests<br>☐ Medical treatment dose adjustment<br>☐ Recommendations for low protein diet and diet calculation<br>☐ Pedagogical support<br>☐ Other (please explain) …………...…………...……………...
25. If provided, what other services would you like to receive from the Metabolism Center?	Please explain:<br>…………...…………...…...………….………….…...………<br>…………...…………...…...………….………….…...………<br>…………...…………...…...………….………….…...………<br>…………...…………...…...………….………….…...………
26. What options do you use to control phenylalanine?<br><br>(*If there is more than one option suitable for you, you can tick it.*)	☐ Phenylalanine-restricted diet<br>☐ Dietary products for medical purposes<br>☐ Large Neutral Amino Acid (LNAA)<br>☐ Sapropterin<br>☐ Other (please explain) …………...…………...………………...

27. What are the difficulties you encounter in applying a phenylalanine restricted diet?<br><br>(*If there is more than one option suitable for you, you can tick it.*)	☐ There are few options available<br>☐ It is difficult to prepare / lack of recipe information<br>☐ It has little/limited effect on reducing phenylalanine levels<br>☐ Reaction from the environment due to diet<br>☐ Diet is causing difficulties at school<br>☐ Lack of taste and flavor<br>☐ Inaccessibility of products due to their expensiveness<br>☐ Social difficulties in adapting to the diet due to reasons such as working life, education, travel (Indicate the social difficulty you experience)<br>……………………………………………………………<br>……………………………………………………………<br>…………………………………………………………… .<br>☐ Other (please explain)<br>……………………………………………………………<br>……………………………………………………………<br>……………………………………………………<br>☐ No, there is none
28. What are the difficulties you encounter in the use of dietary products for medical purposes?<br><br>(If there is more than one option suitable for you, you can tick it.)	☐ There are few options available<br>☐ They have little/limited effect on reducing phenylalanine levels<br>☐ Lack of taste and flavor<br>☐ Being expensive<br>☐ Social difficulties in adapting to the diet due to reasons such as working life, education, travel (Indicate the social difficulty you experience)<br>……………………………………………………………<br>……………………………………………………………<br>……………………………………………………………<br>……………………………………………………………<br>☐ Other (please explain) …………………………………………
29. What difficulties do you face in using Amino Acid or LNAA?<br><br>(If there is more than one option suitable for you, you can tick it.)	☐ They have bad taste<br>☐ They have little/limited effect on reducing phenylalanine levels<br>☐ Difficulty of use due to the large number of tablets<br>☐ Other (please explain)<br>…………………………………………
30. Has your child undergone Sapropterin sensitivity testing for the treatment of PKU?	☐ No (*Go to question 31*)<br>☐ Yes (*Go to question 32*)
31. State the reason why it was not done:	……………………………………………………………<br>……………………………………………………………<br>……………………………………………………………<br>(*Go to question 34.*)
32. If the sapropterin sensitivity test was positive, were you prescribed this medicine and allowed to use it?	☐ Yes<br>☐ No<br>Please state the reason:<br>……………………………………………<br>……………………………………………………………

33. Are there any difficulties you encounter while applying sapropterin treatment? If so, what are they?<br><br>(If there is more than one option suitable for you, you can tick it.)	☐ Volunteer is not sensitive to Sapropterin<br>☐ It has little/limited effect on reducing phenylalanine levels<br>☐ Side effects<br>☐ Other (please explain) …………..…………..………..….<br>☐ NONE	
34. Do you take your child to different specialists for control?<br><br>If your answer is YES, can you please write which department you visited and how many times a year?	☐ Yes (*Go to question 35*)<br>☐ No (*Go to question 36*)	
35. Which specialists do you take your child for control and how many times a year?	☐ Psychiatrist: ………………….times/year<br>☐ Neurologist: ……………………times/year<br>☐ Other (please explain) …………..…………..…………...	
36. Do you have difficulty accessing the metabolism center?	☐ Yes (*Go to question 37*)<br>☐ No (*Go to question 38*)	
37. What difficulties do you experience in accessing the metabolism center?<br><br>(If there is more than one option suitable for you, you can tick it.)	☐ Metabolism center is located in another city<br>☐ Since the center is locatedin another city, it is necessary to stay the next day for test results and treatment dose adjustment.<br>☐ Having difficulties in getting an appointment<br>☐ Long waiting period for examination and control<br>☐ Economic hardship<br>☐ Other (please explain) …………..…………..………..….	
38. Through which institution do you check your child's blood phenylalanine level?	☐ Metabolism center<br>☐ Public Hospital<br>☐ Private hospital<br>☐ Private laboratory<br>☐ Other (please explain) …………..…………..………..….	
39. How often do you go for checking your child's blood phenylalanine level?	☐ Once a year<br>☐ Every 6 months<br>☐ Every 3 months	☐ Monthly<br>☐ Twice a month<br>☐ Other (please explain)<br>……..…………………..
40. What is the target phenylalanine level determined by your doctor or dietitian so that your child can use her/his brain power?	☐ 240 µmol/L<br>☐ 360 µmol/L<br>☐ 480 µmol/L	☐ 600 µmol/L<br>☐ Other (please explain)<br>……..…………………..
41. How many months ago was your child's last blood phenylalanine measurement?	☐ 1 month ago<br>☐ 2 months ago	☐ 3 months ago<br>☐ Other (please explain)<br>……..…………………..
42. What is your child's last blood phenylalanine level?	Please specify: ……… µmol/L	

43. The topics that the patient relatives feel lacking and/or desired to have more information about?<br><br>(If there is more than one option suitable for you, you can tick it.)	☐ Campaigns for disease awareness<br>☐ Patient societies<br>☐ Novel therapies<br>☐ New dietary opportunities (such as recipes)<br>☐ Food contents<br>☐ Group activities for patients and patient relatives<br>☐ More frequent contact with doctor<br>☐ Other (please explain) …………...…………...………………
44. What are the OTHER OPPORTUNITIES that the patient's relative would like to have?<br><br>(If there is more than one option suitable for you, you can tick it.)	☐ Providing the patient families with educational opportunity on PKU management<br>☐ Activation of more metabolism centers for ease of access<br>☐ Opportunity of sending blood samples to metabolism center for phenylalanine measurement<br>☐ Opportunity of control/follow-up without visiting a hospital (control via phone-call and internet /follow-up system, blood collection at home, etc.)<br>☐ More active patient society<br>☐ Awareness activities in every province<br>☐ Availability of home devices for phenylalanine measurement<br>☐ Availability of more options for drug therapy<br>☐ More options for low-protein foods<br>☐ Raising awareness about phenylketonuria in the society<br>☐ Programs about PKU and newborn screening on television/TV series.<br>☐ Other (please explain) ………..…………...………………

III. CHARACTERISTICS RELATED TO SOCIAL LIFE		
45. Are there any positive/negative symptoms your child experience in the **CURRENT SITUATION**? If so, what are they?<br><br>(If there is more than one option suitable for you, you can tick it.)	☐ Failure to concentrate / difficulty in focusing while listening to the teacher<br>☐ Difficulty in understanding the subjects in class<br>☐ Angry/irritable mood<br>☐ Anxious mood<br>☐ Sad mood<br>☐ Rebellious mood<br>☐ Laziness or feeling of laziness<br>☐ Feeling of tiredness<br>☐ Indecisive mood<br>☐ Slow reaction<br>☐ Feeling like in a smokescreen<br>☐ Difficulty in communicating with others<br>☐ Headache<br>☐ Vision impairment<br>☐ Gastric complaints<br>☐ Other (please explain) ……….……………...…………<br>☐ **NONE**	
46. Was there anything your child would like to do but couldn't do because of her/his illness?	☐ Yes, there was (*Go to question 47.*)<br>☐ No there wasn't (*Go to question 48.*)	
47. Write down the thing or things your child wants to do but cannot do due to her/his illness.	1) ……………………………………………………………<br>2) ……………………………………………………………<br>3) ……………………………………………………………<br>4) ……………………………………………………………<br>5) ……………………………………………………………	
48. Is your child's illness a barrier for his/her social life?	☐ Yes	☐ No
49. How often does your child get angry?	☐ Very often<br>☐ Often	☐ Rarely<br>☐ Never
50. Has your child ever been subjected to any bad events due to his/her inability to control his/her anger?	☐ Yes	☐ No
51. How often does your child feel unhappy?	☐ Very often<br>☐ Often	☐ Rarely<br>☐ Never
52. Does your child experience forgetfulness?	☐ Yes (*Go to question 53.*)<br>☐ No (*Go to question 54.*)	
53. How often does he/she experience forgetfulness?	☐ Very often<br>☐ Often	☐ Rarely
54. Do you have any other living children who do not have PKU?	☐ Yes (*Answer questions 55 and 56.*)<br>☐ No (Go to section **IV. Comorbid Chronic Diseases Information)**	

55. What are the difficulties you experience when comparing your healthy child with your child with PKU?	....
56. Is there any difference in the success of your healthy child and your child with PKU in classes and exams?	☐ YES, there is a difference. My child with PKU has lower success rates<br>☐ NO there is no difference.<br>☐ YES, there is a difference. My child with PKU has a higher success rate

IV. INFORMATION ON COMORBID CHRONIC DISEASES

57. Please tick the disease(s) listed on the side that your child has.<br><br>If your child has more than one disease, put a check mark next to each disease.	☐ Myocardial infarct (previous heart attack)<br>☐ Heart failure<br>☐ Peripheral vascular disease (varicose veins, vascular occlusion))<br>☐ Cardiovascular disease<br>☐ Dementia<br>☐ Chronic lung disease (asthma, COPD, tuberculosis)<br>☐ Rheumatic disease (rheumatism)<br>☐ Peptic ulcer (ulcer)<br>☐ Liver disease<br>☐ Diabetes<br>☐ Hemiplegia or paraplegia (partial or complete paralysis)<br>☐ Renal disease<br>☐ Malignancy (cancer)<br>☐ AIDS/HIV
