# Supplementary material for: Phenylketonuria from the perspectives of patients in Türkiye
Source: Orphanet J Rare Dis. 2024 Feb 20;19:78. doi: 10.1186/s13023-024-03079-z (PMC10880278; doi:10.1186/s13023-024-03079-z)
Supplement: Supplementary file 2 — Additional file 2: The adolescent data collection form in Turkish and English. [file 13023_2024_3079_MOESM2_ESM.pdf]

Fenilketonüri (PKU) Hasta Yolculuğu Araştırması

Veri Toplama Formu

Ergenlik Çağı (13-19 Yaş)

ANKETÖR (isminizin yanına çarpı işareti koyunuz)

S.D. ☐

G.Ç. ☐

Hasta Ad/Soyadı Baş Harfleri **Ebeveynin Adı/Soyadı Baş Harfleri**

....

Anket doldurma Tarihi ve Saat

....

AÇIKLAMALAR

Klasik Fenilketonüri tanısı almış ve hakkında bilgi toplanan ergen yaştaki kişi "***Çocuğunuz***", ergen çocuğunuzun bu soru formuna yanıt veren ebeveynleri ise "***Yakını***" olarak tanımlanmaktadır.

GÖNÜLLÜ SEÇİM ÖLÇÜTLERİ

ÇALIŞMAYA DAHİL ETME ÖLÇÜTLERİ

	Evet	Hayır
1. Klasik Fenilketonüri tanısı almış ergenlik çağındaki (13-19) yaş arası çocuklar	☐	☐
2. Çalışma hakkında bilgilendirilen ve yazılı olur veren hasta yakını	☐	☐
3. Araştırma kapsamında kullanılacak veri toplama formunda bulunan sorulara ve değerlendirmelere yanıt vermek için entelektüel kapasiteye sahip olan hasta yakını	☐	☐

Çocuğun çalışmaya alınabilmesi için yukarıdaki soruların tümünün cevabı **E V E T** olmalıdır!

ÇALIŞMA DIŞI BIRAKMA ÖLÇÜTLERİ

	Evet	Hayır
1. Çalışmaya katılmayı istemeyenler.	☐	☐

Çocuğun çalışmaya alınabilmesi için yukarıdaki soruların tümünün cevabı **H A Y I R** olmalıdır!

I. ÇOCUĞUNUZA AİT TANIMLAYICI BİLGİLER

1. Doğum Tarihi	.... / .... / ....<br>Gün Ay Yıl
2. Cinsiyeti	☐ Erkek ☐ Kız
3. Yaşadığı İl	…………………………………………………………
4. Son Mezun Olduğu Okul	…………………………………………………………
5. Çocuğunuzun sağlığı ile evde yaşayanlardan kim ilgileniyor	☐ Anne<br>☐ Kardeş<br>☐ Baba<br>☐ Diğer (açıklayınız): ………………………………………………
6. Hane halkının aylık ortalama geliri	☐ Asgari Ücret seviyesi (2.324 TL)<br>☐ 2.500 - 5.000 TL arası<br>☐ 5.001 - 10.000 TL arası<br>☐ 10.001 – 15.000 TL arası<br>☐ 15.001 TL üstü

**II. METABOLİZMA MERKEZE AİT BİLGİLER**	
7. Sürekli olarak kontrole götürdüğünüz Metabolizma Merkezinin adı nedir?	☐ İstanbul Üniversitesi Tıp Fakültesi - İstanbul<br>☐ Cerrahpaşa Tıp Fakultesi - İstanbul<br>☐ Okmeydanı Eğitim ve Araştırma Hastanesi - İstanbul<br>☐ Marmara Üniversitesi Tıp Fakültesi - İstanbul<br>☐ Hacettepe Üniversitesi Tıp Fakültesi - Ankara<br>☐ Ankara Üniversitesi Tıp Fakültesi - Ankara<br>☐.Gazi Üniversitesi Tıp Fakültesi - Ankara<br>☐.Başkent Üniversitesi Çocuk Hastanesi - Ankara<br>☐.Ankara Şehir Hastanesi - Ankara<br>☐ Dokuz Eylül Üniversitesi Tıp Fakültesi - İzmir<br>☐ Ege Üniversitesi Tıp Fakültesi - İzmir<br>☐ Akdeniz Üniversitesi Tıp Fakültesi - Antalya<br>☐ Antalya Eğitim ve Araştırma Hastanesi - Antalya<br>☐ Çukurova Üniversitesi Tıp Fakültesi - Adana<br>☐ Adana Şehir Hastanesi - Adana<br>☐ Uludağ Üniversitesi Tıp Fakültesi - Bursa<br>☐ Van Eğitim Araştırma hastanesi - Van<br>☐ Diğer ……………………………….……<br><br>☐ **HİÇ BİR** Metabolizma Merkezine kontrole götürmüyoruz gitmiyoruz<br>(*8. soruya geçiniz*)
8. Çocuğunuzu "son iki yılda" kontrol için kaç kez Metabolizma Merkezine götürdünüz?	☐ 1 ☐ 2<br>☐ 3 ☐ 4<br>☐ Daha fazla (belirtiniz): ……….
9. Çocuğunuzu "son bir yılda" kontrol için kaç kez Metabolizma Merkezine götürdünüz?	☐ 1 ☐ 2<br>☐ 3 ☐ 4<br>☐ Daha fazla (*belirtiniz*): ……….………….…………...
10. Halen çocukluk çağında kontrole gittiğiniz Metabolizma Merkezine mi gidiyorsunuz?	☐ **EVET**, hâlâ aynı Metabolizma Merkezine kontrole gidiyoruz *(11. soruya geçiniz)*<br>☐ **HAYIR** farklı bir Metabolizma Merkezine kontrole gidiyoruz *(12. soruya geçiniz)*<br>☐ **HİÇ BİR** Metabolizma Merkezine kontrole götürmüyoruz *(13. soruya geçiniz)*
11. Aynı Metabolizma Merkezine ne sıklıkla gidiyorsunuz?	☐ Son üç yıldır **her yıl İKİ KEREDEN FAZLA** sayıda kontrole gidiyoruz<br>☐ Son üç yıldır **her yıl EN AZ BİR KERE** kontrole gidiyoruz<br>☐ Diğer (*açıklayınız*): …….……..………..…..……….. (*14. soruya geçiniz*)
12. Farklı bir Metabolizma Merkezine ne sıklıkla gidiyorsunuz?	☐ Son üç yıldır **her yıl İKİ KEREDEN FAZLA** sayıda kontrole gidiyoruz<br>☐ Son üç yıldır **her yıl EN AZ BİR KERE** kontrole gidiyoruz<br>☐ Diğer (*açıklayınız*): …….……..………..…..……….. (*14. soruya geçiniz*)

13. Metabolizma Merkezine neden gitmiyorsunuz?	☐ İhtiyaç duymadık<br>☐ Gitmemiz gerektiğini kimse söylemedi<br>☐ İmkanımız yok<br>☐ Diğer (*açıklayınız*): ………….…………..…………..<br>(*14. soruya geçiniz*)
14. İmkanınız olsa kontrole gittiğiniz Metabolizma Merkezi ya da Metabolizma Uzmanını değiştirmek ister miydiniz?	☐ Evet, değiştirmek isterdim.<br>(*15. soruya geçiniz*)
	☐ Hayır, değiştirmek istemezdim.<br>(*16. soruya geçiniz*)
15. Nedenini belirtiniz	…….……………….……….…………………….……
16. Düzenli olarak **<u>diyetisyen kontrolüne</u>** gidiyor musunuz?	☐ Evet düzenli olarak gidiyoruz (*17. soruya geçiniz*)
	☐ Hayır düzenli olarak gitmiyoruz (*17. soruya geçiniz*)
	☐ Hiçbir diyetisyene kontrole gitmiyoruz. (*18. soruya geçiniz*)
17. Diyetisyene ne sıklıkla kontrole gidiyorsunuz?	☐ **Her yıl İKİ KEREDEN FAZLA** sayıda kontrole gidiyoruz<br>☐ **Her yıl EN AZ BİR KERE** kontrole gidiyoruz<br>☐ Diğer (*açıklayınız*): ………….…………..…………..
18. Diyetisyene neden gitmiyorsunuz?	☐ İhtiyaç duymadık<br>☐ Gitmemiz gerektiğini kimse söylemedi<br>☐ Diyeti kendi isteğimizle bıraktık<br>☐ Diyeti bırakmamız söylendi<br>☐ Tedavi bırakıldı<br>☐ İmkanımız yok<br>☐ Gerekli olmadığını düşünüyoruz<br>☐ Diğer (*açıklayınız*): ………….…………..…………..
19. Çocuğunuzu çocukluk çağında hiç **<u>Pedagoga</u>** götürdünüz mü?	☐ **Evet** (*20. soruya geçiniz*)
	☐ **Hayır** (*23. soruya geçiniz*)
20. Çocukluk çağında gittiği Pedagoga hala gidiyor musunuz?	☐ Evet aynı Pedagoga gidiyoruz (*21. soruya geçiniz*)
	☐ Hayır farklı bir Pedagoga gidiyoruz (*22. soruya geçiniz*)
	☐….. kere gittik, ama artık gitmiyoruz. (*23. soruya geçiniz*)
21. Aynı Pedagoga ne sıklıkla gidiyorsunuz?	☐ **Her yıl İKİ KEREDEN FAZLA**<br>☐ **Her yıl EN AZ BİR KERE**<br>☐ Diğer
22. Farklı Pedagoga ne sıklıkla gidiyorsunuz?	☐ **Her yıl İKİ KEREDEN FAZLA**<br>☐ **Her yıl EN AZ BİR KERE**<br>☐ Diğer
23. Pedagoga neden gitmiyorsunuz?	☐ İhtiyaç duymadık.<br>☐ Gitmemiz gerektiğini kimse söylemedi.<br>☐ İmkanımız yok.<br>☐ Gerekli olmadığını düşünüyoruz.<br>☐ Diğer………………

24. Şu anda hizmet aldığınız Metabolizma Merkezinden memnuniyet durumunuz nedir?	☐ Çok memnunuz<br>☐ Memnunuz<br>☐ Memnun değiliz (*Nedenini belirtiniz*):<br>……………………………………………………………<br>……………………………………………………………
25. Şu anda hizmet aldığınız Metabolizma Uzmanından memnuniyet durumunuz nedir?	☐ Çok memnunuz<br>☐ Memnunuz<br>☐ Memnun değiliz (*Nedenini belirtiniz*):<br>……………………………………………………………<br>……………………………………………………………
26. Metabolizma Merkezinde aldığınız hizmetler nelerdir?	☐ Fenilalanin kan seviyesi ölçümü<br>☐ Diğer laboratuvar testleri<br>☐ Tıbbi tedavi doz ayarlaması<br>☐ Düşük proteinli Beslenme ve diyet hesaplaması önerileri<br>☐ Pedagog desteği<br>☐ Diğer (*açıklayınız*): ……………………………………..
27. Eğer veriliyor olsaydı Metabolizma Merkezinden almak istediğiniz diğer hizmetler neler olurdu?	Açıklayınız:<br>……………………………………………………………<br>……………………………………………………………<br>……………………………………………………………<br>……………………………………………………………
28. Fenilalanin kontrolü için kullandığınız seçenekler hangileridir?<br><br>(*sizin için uygun birden fazla seçenek var ise işaretleyebilirsiniz.*)	☐ Fenilalanin kısıtlı diyet<br>☐ Tıbbi amaçlı diyet ürünleri<br>☐ Amino asitler<br>☐ Büyük Nötral Aminoasit (LNAA)<br>☐ Sapropterin<br>☐ Diğer (*açıklayınız*): ………………………………………...
29. Fenilalanin kısıtlı diyet uygulamasında karşılaştığınız zorluklar nelerdir?<br><br>(*sizin için uygun birden fazla seçenek var ise işaretleyebilirsiniz.*)	☐ Seçeneklerin az olması<br>☐ Hazırlamanın zor olması / tarif bilgilerinin olmaması<br>☐ Fenilalanin seviyesini düşürmede etkisinin az/kısıtlı olması<br>☐ Beslenme şekli sebebiyle çevreden tepki alması<br>☐ Beslenme şeklinin okulda zorluklara sebep olması<br>☐ Tad ve lezzet eksikliği<br>☐ Ürünlerin pahalı olması nedeniyle erişilememesi<br>☐ Çalışma hayatı, eğitim, seyahat gibi nedenlerle diyete uyumun sosyal zorlukları (*Yaşadığınız sosyal zorluğu belirtiniz*)<br>……………………………………………………………<br>……………………………………………………………<br>……………………………………………………………<br>……………………………………………………………<br>☐ Diğer (*açıklayınız*):<br>……………………………………………………………<br>……………………………………………………………<br>……………………………………………………………<br>……………………………………………………………<br>☐ Hayır, yok

30. Düşük Proteinli ürünlerin kullanımında karşılaştığınız zorluklar nelerdir?<br><br>(sizin için uygun birden fazla seçenek var ise işaretleyebilirsiniz.)	☐ Seçeneklerin az olması<br>☐ Fenilalanin seviyesini düşürmede etkisinin az/kısıtlı olması<br>☐ Tad ve lezzet eksikliği<br>☐ Pahalı olması<br>☐ Çalışma hayatı, eğitim, seyahat gibi nedenlerle diyete uyumun sosyal zorlukları (*Yaşadığınız sosyal zorluğu belirtiniz*)<br>………………………………………………………………<br>………………………………………………………………<br>………………………………………………………………<br>………………………………………………………………<br>☐ Diğer (*açıklayınız*): ………………………………………...
31. Amino Asit veya LNAA kullanımında karşılaştığınız zorluklar nelerdir?<br><br>(sizin için uygun birden fazla seçenek var ise işaretleyebilirsiniz.)	☐ Tatlarının kötü olması<br>☐ Fenilalanin seviyesini düşürmede etkisinin az/kısıtlı olması<br>☐ Tablet sayısının fazla olması nedeniyle kullanım güçlüğü<br>☐ Diğer (*açıklayınız*): …………………………………………
32. Çocuğunuza PKU tedavisi için Sapropterin duyarlılık testi yapıldı mı?	☐ Hayır (*33. soruya geçiniz*)<br>☐ Evet (*34. soruya geçiniz*)
33. Yapılmamasının nedenini belirtiniz	………………………………………………………………<br>………………………………………………………<br>………………………………………………………………<br>(*36. soruya geçiniz.*)
34. Sapropterin duyarlılık testi pozitif çıktıysa size bu ilaç reçete edilerek kullanmanız sağlandı mı?	☐ Evet (*35. soruya geçiniz.*)<br>☐ Hayır<br>Nedenini belirtiniz: ………………………………………………..<br>………………………………………………………………..<br>(*36. soruya geçiniz.*)
35. Sapropterin tedavisi uygularken karşılaştığınız zorluklar var mı, varsa nelerdir?<br><br>(sizin için uygun birden fazla seçenek var ise işaretleyebilirsiniz.)	☐ Gönüllünün Sapropterin duyarlı olmaması<br>☐ Fenilalanin seviyesini düşürmede etkisinin az/kısıtlı olması<br>☐ Yan etkileri<br>☐ Diğer (*açıklayınız*): …………………………………………<br>☐ YOK
36. Çocuğunuzu kontrol için farklı bölüm uzmanlarına götürüyor musunuz?<br>Cevabınız EVET ise hangi bölüme - yılda kaç kez gittiğinizi yazar mısınız?	☐ Evet (*37. soruya geçiniz*)<br>☐ Hayır (*38. soruya geçiniz*)
37. Çocuğunuzu kontrol için hangi uzmanlara, yılda kaç kez götürüyorsunuz?	☐ Psikiyatrist: ………………….kez/yıl<br>☐ Nörolog: ……………………kez/yıl<br>☐ Diğer (*açıklayınız*): …………………………………………
38. Metabolizma merkezine erişimde zorluk yaşıyor musunuz?	☐ Evet (*39. soruya geçiniz*)<br>☐ Hayır (*40. soruya geçiniz*)

39. Metabolizma merkezine erişimde ne tür zorluklar yaşıyorsunuz?<br><br>(sizin için uygun birden fazla seçenek var ise işaretleyebilirsiniz.)	☐ Metabolizma merkezinin başka şehirde olması<br>☐ Merkezin başka şehirde olması nedeniyle test sonuçları ve tedavi doz ayarlaması için ertesi gün konaklama yapma zorunluluğu olması<br>☐ Randevu almanın zorlukları olması<br>☐ Muayene ve kontrol için bekleme süresinin çok uzun olması<br>☐ Ekonomik zorluklar olması<br>☐ Diğer (*açıklayınız*): ………….………….……………..….	
40. Çocuğunuzun kan fenilalanin değeri kontrolünü hangi kurum aracılığı ile sağlıyorsunuz	☐ Metabolizma merkezi<br>☐ Devlet hastanesi<br>☐ Özel hastane<br>☐ Özel laboratuvar<br>☐ Diğer (*açıklayınız*): ………….………….……………..….	
41. Çocuğunuzun kan fenilalanin değerini ne sıklıkla takip ediyorsunuz?	☐ Yılda bir<br>☐ 6 ayda bir<br>☐ 3 ayda bir	☐ Ayda bir<br>☐ Ayda iki<br>☐ Diğer (*açıklayınız*):<br>…………..…………………
42. Doktorunuzun ya da diyetisyenin, çocuğunuzun, beyin gücünü kullanabilmesi için belirlediği hedef fenilalanin düzeyi nedir?	☐ 240 µmol/L<br>☐ 360 µmol/L<br>☐ 480 µmol/L	☐ 600 µmol/L<br>☐ Diğer (*açıklayınız*):<br>…………..…………………
43. Çocuğunuzun kan fenilalanin ölçümü en son kaç ay önce yapıldı?	☐ 1 ay önce<br>☐ 2 ay önce	☐ 3 ay önce<br>☐ Diğer (*açıklayınız*):<br>…………..…………………
44. Çocuğunuzun en son ölçülen kan fenilalanin değeri nedir?	Belirtiniz: ……… µmol/L	
45. Hasta yakınının daha çok bilgi sahibi olmayı da bilgilendirilmeyi istediği konular nelerdir?<br><br>(sizin için uygun birden fazla seçenek var ise işaretleyebilirsiniz.)	☐ Hastalık farkındalığı kampanyaları<br>☐ Hasta dernekleri<br>☐ Yeni tedaviler<br>☐ Yeni beslenme olanakları (yemek tarifleri gibi)<br>☐ Gıda içerikleri<br>☐ Hasta ve yakınlarına ait grup aktiviteleri<br>☐ Doktor ile daha sık görüşmek<br>☐ Diğer (*açıklayınız*): ………….………….……………..….	

46. Hasta yakınının sahip olmak istediği **DİĞER İMKANLAR** nelerdir?<br><br>(sizin için uygun birden fazla seçenek var ise işaretleyebilirsiniz.)	☐ Hasta ailelerine PKU yönetimi konusunda eğitim olanaklarının sağlanması<br>☐ Erişim kolaylığı için daha fazla Metabolizma Merkezinin faaliyete geçmesi<br>☐ Metabolizma merkezine fenilalanin ölçümü için kan gönderme imkanı olması<br>☐ Hastaneye gitmeden telefon ve internet ile takip ve kontrol sistemi kurulması (evde kan alma, uzaktan fenilalanin ve uzman kontrolü)<br>☐ Hasta Derneğinin daha aktif olması<br>☐ Her ilde farkındalık faaliyetlerinin yapılması<br>☐ Fenilalanin seviyesinin ölçülmesi için Evde ölçüm cihazlarının bulunması<br>☐ Daha fazla ilaç tedavisi seçeneği olması<br>☐ Daha fazla düşük proteinli gıda seçeneği olması<br>☐ Toplumda fenilketonüri konusunda farkındalık yaratılması<br>☐ TV de, dizilerde PKU, yenidoğan taraması anlatılması.<br>☐ Diğer (*açıklayınız*): ………….…………..……………..….

**III. SOSYAL YAŞAMA AİT ÖZELLİKLER**		
47. Çocuğunuzun **MEVCUT DURUMDA** yaşadığı olumlu / olumsuz bulgular var mı? Var ise nelerdir?<br><br>(sizin için uygun birden fazla seçenek var ise işaretleyebilirsiniz.)	☐ Ders dinlerken dikkat toplayamama / odaklanma güçlüğü<br>☐ Dersi anlamada güçlük çekme<br>☐ Öfkeli / sinirli ruh hali<br>☐ Endişeli ruh hali<br>☐ Üzüntülü ruh hali<br>☐ İsyankar ruh hali<br>☐ Tembellik ya da tembellik hissi<br>☐ Yorgunluk hissi<br>☐ Kararsız ruh hali<br>☐ Reaksiyon yavaşlığı<br>☐ Sis perdesi içinde olduğunu hissetmek<br>☐ Başkaları ile İletişim güçlüğü<br>☐ Baş ağrısı<br>☐ Görme bozukluğu<br>☐ Mide şikayetleri<br>☐ Diğer (*açıklayınız*): …………..………..…………<br>☐ **YOK**	
48. Çocuğunuzun hastalığı sebebiyle yapmak isteyip yapamadığı bir şey oldu mu?	☐ Evet oldu (*49. soruya geçiniz.*)<br>☐ Hayır olmadı (*50. soruya geçiniz.*)	
49. Çocuğunuzun hastalığı sebebiyle yapmak isteyip yapamadığı şeyi veya şeyleri yazınız.	1) ……………………………………………………………<br>2) ……………………………………………………………<br>3) ……………………………………………………………<br>4) ……………………………………………………………<br>5) ……………………………………………………………	
50. Çocuğunuzun hastalığı sosyal yaşantısı için bir engel mi?	☐ Evet	☐ Hayır
51. Çocuğunuz ne kadar sıklıkla öfkeleniyor?	☐ Çok sık<br>☐ Sık	☐ Nadiren<br>☐ Hiç
52. Çocuğunuz öfkesini kontrol edememesi nedeniyle herhangi bir kötü olaya maruz kaldı mı?	☐ Evet	☐ Hayır
53. Çocuğunuz ne kadar sıklıkla kendisini mutsuz hissediyor?	☐ Çok sık<br>☐ Sık	☐ Nadiren<br>☐ Hiç
54. Çocuğunuz unutkanlık yaşıyor mu?	☐ Evet (*55. soruya geçiniz.*)<br>☐ Hayır (*56. soruya geçiniz.*)	
55. Ne sıklıkla unutkanlık yaşıyor?	☐ Çok sık<br>☐ Sık	☐ Nadiren

56. PKU hastası olmayan yaşayan başka çocuğunuz var mı?	☐ Evet (*57. ve 58. soruyu yanıtlayınız.*)<br>☐ Hayır (**IV. Eşlik Eden Kronik Hastalık Bilgileri** bölümüne geçiniz.)
57. Sağlıklı çocuğunuzla PKU hastası çocuğunuz karşılaştırdığınızda yaşadığınız zorluklar nelerdir?	……………………………………………………………<br>……………………………………………………………<br>……………………………………………………………
58. Sağlıklı çocuğunuzla PKU hastası çocuğunuzun dersleri ve sınavlarındaki başarıları farklılık gösteriyor mu?	☐ EVET farklılık var. PKU hastası çocuğumun başarısı daha düşük<br>☐ HAYIR farklılık yok.<br>☐ EVET farklılık var. PKU hastası çocuğumun başarısı daha yüksek

**IV. EŞLİK EDEN KRONİK HASTALIK BİLGİLERİ**	
59. Çocuğunuzda bulunan ve yan tarafta listelenen hastalık ya da hastalıkları işaretleyiniz.<br><br>Çocuğunuzda birden fazla hastalığın olması halinde ilgili her hastalığın yanına işaret koyunuz.	☐ Miyokart enfarktüsü (geçirilmiş kalp krizi)<br>☐ Kalp yetmezliği<br>☐ Periferik damar hastalığı (varis, damar tıkanıklığı)<br>☐ Kalp-damar hastalığı<br>☐ Demans (bunama)<br>☐ Kronik akciğer hastalığı (astım, KOAH, tüberküloz)<br>☐ Romatizmal hastalık (romatizma)<br>☐ Peptik ülser (ülser)<br>☐ Karaciğer hastalığı<br>☐ Diyabet (şeker hastalığı)<br>☐ Hemipleji ya da parapleji (kısmı ya da tam felç)<br>☐ Böbrek hastalığı<br>☐ Malignite (kanser hastalığı)<br>☐ AIDS/HIV

V. ERGEN ÇOCUĞA SORULACAK SORULAR

60. Yan tarafta listelenen durumlardan senin için geçerli olanları belirtmeni istiyoruz.<br><br>(senin için uygun birden fazla seçenek var ise işaretleyebilirsin)	☐ Dikkat toplayamama / odaklanma güçlüğü<br>☐ İş ve gündelik hayatta konuları anlamada güçlük çekme<br>☐ Öfkeli / sinirli ruh hali<br>☐ Endişeli ruh hali<br>☐ Üzüntülü ruh hali<br>☐ İsyankar ruh hali<br>☐ Tembellik ya da tembellik hissi<br>☐ Yorgunluk hissi<br>☐ Kararsız ruh hali<br>☐ Reaksiyon yavaşlığı<br>☐ Sis perdesi içinde olduğunu hissetmek<br>☐ Başkaları ile iletişim güçlüğü<br>☐ Baş ağrısı<br>☐ Görme bozukluğu<br>☐ Mide şikayetleri<br>☐ Diğer (*açıklayınız*): …………..…………..………….<br>☐ **YOK**	
61. Hastalığın sebebiyle yapmak isteyip yapamadığın bir şey oldu mu?	☐ Evet oldu (*62. soruya geçiniz.*)<br>☐ Hayır olmadı (*63. soruya geçiniz.*)	
62. Yapmak isteyip yapamadığın şeyi veya şeyleri yazar mısın?	1) ……………………………………………………………<br>2) ……………………………………………………………<br>3) ……………………………………………………………<br>4) ……………………………………………………………<br>5) ……………………………………………………………	
63. Hastalığın sosyal yaşantın için bir engel mi?	☐ Evet	☐ Hayır
64. Ne kadar sıklıkla öfkeleniyorsun?	☐ Çok sık<br>☐ Sık	☐ Nadiren<br>☐ Hiç
65. Öfkeni kontrol edememen nedeniyle herhangi bir kötü olaya maruz kaldın mı?	☐ Evet	☐ Hayır
66. Ne kadar sıklıkla kendini mutsuz hissediyorsun?	☐ Çok sık<br>☐ Sık	☐ Nadiren<br>☐ Hiç
67. Unutkanlık yaşıyor musun?	☐ Evet (*68. soruya geçiniz.*)<br>☐ Hayır (*69. soruya geçiniz.*)	
68. Ne sıklıkla unutkanlık yaşıyorsun?	☐ Çok sık<br>☐ Sık	☐ Nadiren
69. Eğitim hayatında zorluklarla karşılaşıyor musun?	☐ Evet (*70. soruya geçiniz.*)<br>☐ Hayır (*71. soruya geçiniz.*)	

70. Eğitim hayatında karşılaştığın zorlukları yazar mısın?	1) ....<br>2) ....<br>3) ....<br>4) ....<br>5)	
71. Planlama yaparken zorluk yaşıyor musun?	☐ Evet	☐ Hayır
72. Herhangi bir konuda karar vermen gerektiğinde karar verirken güçlük yaşıyor musun?	☐ Evet yaşıyorum	☐ Hayır yaşamıyorum

Phenylketonuria: The Patient’s Journey Study

Data Collection Form

ADOLESCENT GROUP (Between 13 and 19 Years of Age)

INTERVIEWER (Put a cross next to your name)

S.D ☐

G.Ç. ☐

Initials of the Patient’s Name/Surname **Initials of the Parent's Name/Surname**

....

Survey Completion Date and Time

....

EXPLANATIONS

The person in the adolescence age who has been diagnosed with Classical Phenylketonuria and about whom information is collected is defined as **"*Your Child*",** and you, the parents of your child who responded to this questionnaire, are defined as **"*Relatives*".**

VOLUNTEER SELECTION CRITERIA

STUDY INCLUSION CRITERIA		
	**Yes**	**No**
1. Children in the adolescence period between 13 and 19 years of age diagnosed with Classical Phenylketonuria	☐	☐
2. The patient's relative who was informed about the study and gave written consent	☐	☐
3. The patient's relative who has the intellectual capacity to answer the questions and evaluations in the data collection form to be used within the scope of the study	☐	☐
In order for the child to be included in the study, the answer to all of the above questions must be **Y E S!**		

STUDY EXCLUSION CRITERIA		
	**Yes**	**No**
1. Those who do not want to participate in the study.	☐	☐
In order for the child to be included in the study, the answer to all of the above questions must be **N O!**		

I. DESCRIPTIVE INFORMATION ABOUT YOUR CHILD

1. Date of Birth	.... / .... / ....<br>Day Month Year	
2. Gender	☐ Boy	☐ Girl
3. City of residence	…………………………………………………………	
4. The last school he/she graduated from	…………………………………………………………	
5. Among the people living at home, who takes care about your child's health?	☐ Mother<br>☐ Sibling<br>☐ Father<br>☐ Other (please explain):<br>………………………………………………	
6. Average monthly income of the household	☐ Minimum Wage level (2.324 TL)<br>☐ Between 2.500 - 5.000 TL<br>☐ Between 5.001 - 10.000 TL<br>☐ Between 10.001 – 15.000 TL<br>☐ Over 15.001 TL	

II. INFORMATION ABOUT THE METABOLISM CENTER	
7. What is the name of the Metabolism Center where you regularly take your child for control?	☐ İstanbul University School of Medicine - İstanbul<br>☐ Cerrahpaşa School of Medicine - İstanbul<br>☐ Okmeydanı Training and Research Hospital - İstanbul<br>☐ Marmara University School of Medicine - İstanbul<br>☐ Hacettepe University School of Medicine - Ankara<br>☐ Ankara University School of Medicine - Ankara<br>☐.Gazi University School of Medicine - Ankara<br>☐.Başkent University Children's Hospital - Ankara<br>☐.Ankara City Hospital - Ankara<br>☐ Dokuz Eylül University School of Medicine - İzmir<br>☐ Ege University School of Medicine - İzmir<br>☐ Akdeniz University School of Medicine - Antalya<br>☐ Antalya Training and Research Hospital - Antalya<br>☐ Çukurova University School of Medicine - Adana<br>☐ Adana City Hospital - Adana<br>☐ Uludağ University School of Medicine - Bursa<br>☐ Van Training and Research Hospital - Van<br>☐ Other ………………………………….……<br><br>☐ **Neither of them** We do not go to a Metabolism Center for control.<br>(*Go to question 8*)
8. How many times have you taken your child to the Metabolism Center for control in the "last two years"?	☐ 1 ☐ 2<br>☐ 3 ☐ 4<br>☐ More (specify)………
9. How many times have you taken your child to the Metabolism Center for control in the "last year"?	☐ 1 ☐ 2<br>☐ 3 ☐ 4<br>☐ More (specify)………
10. Do you still visit the Metabolism Center where you went for control in childhood?	☐ **YES**, we still visit the same Metabolism Center for control. *(Go to question 11)*<br>☐ **NO,** we are visiting a different Metabolism Center for control. *(Go to question 12)*<br>☐ **Neither of them** We do not visit a Metabolism Center for control (*Go to question 13)*
11. How often do you visit the same Metabolism Center?	☐ We have been going for control **MORE THAN TWICE every year** for the last three years.<br>☐ We have been going for control **AT LEAST ONCE a year** for the last three years.<br>☐ Other (please explain): ……………………………….<br>*(Go to question 14)*

12. How often do you go to another Metabolism Center?	☐ We have been going for control **MORE THAN TWICE every year** for the last three years<br>☐ We have been going for control **AT LEAST ONCE a year** for the last three years.<br>☐ Other (please explain) …………...…………...…………...<br>(*Go to question 14*)
13. Why don't you go to a Metabolism Center?	☐ We didn't need to.<br>☐ Nobody told us that we should go<br>☐ We don't have the financial means<br>☐ Other (please explain) …………...…………...…………...<br>(*Go to question 14*)
14. If you had the opportunity, would you want to change the Metabolism Center or Metabolism Specialist you visit for control?	☐ Yes, I would like to change.<br>(*Go to question 15*)
	☐ No, I wouldn't want to change.<br>(*Go to question 16*)
15. Please state the reason	………………………………………………………
16. Do you **visit a dietitian** regularly?	☐ Yes, we go regularly (*Go to question 17*)
	☐ No, we don't go regularly (*Go to question 18*)
	☐ We do not visit any dietician for control. (*Go to question 18*)
17. How often do you visit a dietitian for control?	☐ We are going for control **MORE THAN TWICE every year**<br>☐ We are going for control **AT LEAST ONCE a year**<br>☐ Other (please explain) …………...…………...…………...
18. Why don't you visit a dietician?	☐ We didn't need to<br>☐ Nobody told us that we should go<br>☐ We left the diet voluntarily.<br>☐ We were told to stop dieting<br>☐ Treatment was stopped<br>☐ We don't have the financial means<br>☐ We think it is not necessary<br>☐ Other (please explain) …………...…………...…………...
19. Have you ever taken your child to a **Pedagogue** in childhood?	☐ **Yes** (Go to question *20*)
	☐ **No** (Go to question *23*)
20. Do you still go to the pedagogue that you have taken your child in the childhood?	☐ Yes, we go to the same pedagogue (go to question 21)
	☐ No, we are going to a different pedagogue (go to question 22)
	☐ We went …. times, but we don't go anymore. (Go to question 23)
21. How often do you visit the same pedagogue?	☐ **MORE THAN TWICE every year**<br>☐ **AT LEAST ONCE a year**<br>☐ Other (please explain) …………...…………...…………...

22. How often do you go to a different pedagogue?	☐ **MORE THAN TWICE every year**<br>☐ **AT LEAST ONCE a year**<br>☐ Other ……….
23. Why didn't you visit a Pedagog?	☐ We didn't need to<br>☐ Nobody told us that we should go<br>☐ We don't have the financial means<br>☐ We think it is not necessary<br>☐ Other (please explain) …………...…………...…………...
24. What is your satisfaction level with the Metabolism Center you are currently receiving service from?	☐ We are very satisfied<br>☐ We are satisfied<br>☐ We are not satisfied (specify why)...……………………………………………………………<br>…………………………………………………………………
25. What is your satisfaction level with the Metabolism Specialist you are currently receiving service from?	☐ We are very satisfied<br>☐ We are satisfied<br>☐ We are not satisfied (specify why):<br>…………………………………………………………………<br>…………………………………………………………………
26. What services do you receive from the Metabolism Center?	☐ Phenylalanine blood level measurement<br>☐ Other laboratory tests<br>☐ Medical treatment dose adjustment<br>☐ Recommendations for low protein diet and diet calculation<br>☐ Pedagogical support<br>☐ Other (please explain) …………...…………...…………...
27. If provided, what other services would you like to receive from the Metabolism Center?	Please explain:<br>…………………………………………………………………<br>…………………………………………………………………<br>…………………………………………………………………<br>…………………………………………………………………
28. What options do you use to control phenylalanine?<br><br>(*If there is more than one option suitable for you, you can tick it.*)	☐ Phenylalanine-restricted diet<br>☐ Dietary products for medical purposes<br>☐ Amino acids<br>☐ Large Neutral Amino Acid (LNAA)<br>☐ Sapropterin<br>☐ Other (please explain) …………...…………...…………

29. What are the difficulties you encounter in applying a phenylalanine restricted diet?<br><br>(*If there is more than one option suitable for you, you can tick it.*)	☐ There are few options available<br>☐ It is difficult to prepare / lack of recipe information<br>☐ It has little/limited effect on reducing phenylalanine levels<br>☐ Reaction from the environment due to diet<br>☐ Diet is causing difficulties at school<br>☐ Lack of taste and flavor<br>☐ Inaccessibility of products due to their expensiveness<br>☐ Social difficulties in adapting to the diet due to reasons such as working life, education, travel (Indicate the social difficulty you experience)<br>……………………………………………………………<br>……………………………………………………………<br>……………………………………………………………<br>☐ Other (please explain)<br>……………………………………………………………<br>……………………………………………………………<br>……………………………………………………………<br>☐ No, there is none
30. What are the difficulties you encounter in the use of low-protein products?<br><br>(If there is more than one option suitable for you, you can tick it.)	☐ There are few options available<br>☐ They have little/limited effect on reducing phenylalanine levels<br>☐ Lack of taste and flavor<br>☐ Being expensive<br>☐ Social difficulties in adapting to the diet due to reasons such as working life, education, travel (Indicate the social difficulty you experience)<br>……………………………………………………………<br>……………………………………………………………<br>……………………………………………………………<br>……………………………………………………………<br>☐ Other (please explain)
31. What are the difficulties do you face in using Amino Acid or LNAA?<br><br>(If there is more than one option suitable for you, you can tick it.)	☐ They have bad taste<br>☐ They have little/limited effect on reducing phenylalanine levels<br>☐ Difficulty of use due to the large number of tablets<br>☐ Other (please explain)
32. Has your child undergone Sapropterin sensitivity testing for the treatment of PKU?	☐ No (*Go to question 33*)<br>☐ Yes (*Go to question 34*)
33. State the reason why it was not done:	……………………………………………………………<br>……………………………………………………………<br>……………………………………………………………<br>(*Go to question 36.*)
34. If the sapropterin sensitivity test was positive, were you prescribed this medicine and allowed to use it?	☐ Yes (*Go to question 35*)<br>☐ No<br>Please state the reason<br>……………………………………………<br>……………………………………………………………<br>(*Go to question 36*)

35. Are there any difficulties you encounter while applying sapropterin treatment? If so, what are they?<br><br>(If there is more than one option suitable for you, you can tick it.)	☐ Volunteer is not sensitive to Sapropterin<br>☐ It has little/limited effect on reducing phenylalanine levels<br>☐ Side effects<br>☐ Other (please explain) …………..…………..……….…..<br>☐ NONE	
36. Do you take your child to different specialists for control?<br>If your answer is YES, can you please write which department you visited and how many times a year?	☐ Yes (*Go to question 37*)<br>☐ No (*Go to question 38*)	
37. Which specialists do you take your child for control and how many times a year?	☐ Psychiatrist: ………………….times/year<br>☐ Neurologist: ……………………times/year<br>☐ Other (please explain) …………..…………..…………..….	
38. Do you have difficulty accessing the metabolism center?	☐ Yes (*Go to question 39*)<br>☐ No (*Go to question* 40)	
39. What difficulties do you experience in accessing the metabolism center?<br><br>(If there is more than one option suitable for you, you can tick it.)	☐ Metabolism center is located in another city<br>☐ Since the center is located in another city, it is necessary to stay the next day for test results and treatment dose adjustment.<br>☐ Having difficulties in getting an appointment<br>☐ Long waiting period for examination and control<br>☐ Economic hardship<br>☐ Other (please explain)	
40. Through which institution do you check your child's blood phenylalanine level?	☐ Metabolism center<br>☐ Public Hospital<br>☐ Private hospital<br>☐ Private laboratory<br>☐ Other (please explain) …………..…………..…………..….	
41. How often do you go for the control of your child's blood phenylalanine level?	☐ Once a year<br>☐ Every 6 months<br>☐ Every 3 months	☐ Monthly<br>☐ Twice a month<br>☐ Other (please explain) ……..………………….
42. What is the target phenylalanine level determined by your doctor or dietitian so that your child can use her/his brain power?	☐ 240 µmol/L<br>☐ 360 µmol/L<br>☐ 480 µmol/L	☐ 600 µmol/L<br>☐ Other (please explain) …………..………………….
43. How many months ago was your child's last blood phenylalanine measurement?	☐ 1 month ago<br>☐ 2 months ago	☐ 3 months ago<br>☐ Other (please explain) ……..………………….
44. What is your child's last blood phenylalanine level?	Please specify: ……… µmol/L	

45. The topics that the patient relatives feel lacking and/or desired to have more information about?<br><br>(If there is more than one option suitable for you, you can tick it.)	☐ Campaigns for disease awareness<br>☐ Patient societies<br>☐ Novel therapies<br>☐ New dietary opportunities (such as recipes)<br>☐ Food contents<br>☐ Group activities for patients and patient relatives<br>☐ More frequent contact with doctor<br>☐ Other (please explain) …………..…………..…………..….
46. What are the OTHER OPPORTUNITIES that the patient's relative would like to have?<br><br>(If there is more than one option suitable for you, you can tick it	☐ Providing the patient families with educational opportunity on PKU management<br>☐ Activation of more metabolism centers for ease of access<br>☐ Opportunity of sending blood samples to metabolism center for phenylalanine measurement<br>☐ Opportunity of control/follow-up without visiting a hospital (control via phone-call and internet /follow-up system, blood collection at home, etc.)<br>☐ More active patient society<br>☐ Awareness activities in every province<br>☐ Availability of home devices for phenylalanine measurement<br>☐ Availability of more options for drug therapy<br>☐ More options for low-protein foods<br>☐ Raising awareness about phenylketonuria in the society<br>☐ Programs about PKU and newborn screening on television/TV series.<br>☐ Other (please explain) …………..…………..…………..….

**III. CHARACTERISTICS RELATED TO SOCIAL LIFE**	
47. Are there any positive/negative symptoms your child experience in the **CURRENT SITUATION**? If so, what are they?<br><br>(If there is more than one option suitable for you, you can tick it.))	☐ Failure to concentrate / difficulty in focusing while listening to the teacher<br>☐ Difficulty in understanding the subjects in class<br>☐ Angry/irritable mood<br>☐ Anxious mood<br>☐ Sad mood<br>☐ Rebellious mood<br>☐ Laziness or feeling of laziness<br>☐ Feeling of tiredness<br>☐ Indecisive mood<br>☐ Slow reaction<br>☐ Feeling like in a smokescreen<br>☐ Difficulty in communicating with others<br>☐ Headache<br>☐ Vision impairment<br>☐ Gastric complaints<br>☐ Other (please explain) ………..…………...………..<br>☐ **NONE**

48. Was there anything your child would like to do but couldn't do because of her/his illness?	☐ Yes, there was (*Go to question 49.*)	
	☐ No there wasn't (*Go to question 50.*)	
49. Write down the thing or things your child wants to do but cannot do due to her/his illness.	1) ....<br>2) ....<br>3) ....<br>4) ....<br>5)	
50. Is your child's illness a barrier for his/her social life?	☐ Yes	☐ No
51. How often does your child get angry?	☐ Very often<br>☐ Often	☐ Rarely<br>☐ Never
52. Has your child ever been subjected to any bad events due to his/her inability to control his/her anger?	☐ Yes	☐ No
53. How often does your child feel unhappy?	☐ Very often<br>☐ Often	☐ Rarely<br>☐ Never
54. Does your child experience forgetfulness?	☐ Yes (*Go to question 55.*)	
	☐ No (*Go to question 56.*)	
55. How often does he/she experience forgetfulness?	☐ Very often<br>☐ Often	☐ Rarely
56. Do you have any other living children who do not have PKU?	☐ Yes (*Answer questions 57 and 58*)	
	☐ No (Go to section **IV. Comorbid Chronic Diseases Information)**	
57. What are the difficulties you experience when comparing your healthy child with your child with PKU?	....<br>..<br>..	
58. Is there any difference in the success of your healthy child and your child with PKU in classes and exams?	☐ YES, there is a difference. My child with PKU has lower success rates<br>☐ NO there is no difference.<br>☐ YES, there is a difference. My child with PKU has a higher success rate	

IV. INFORMATION ON COMORBID CHRONIC DISEASES	
59. Please tick the disease(s) listed on the side that your child has.<br><br>If your child has more than one disease, put a check mark next to each disease.	☐ Myocardial infarct (previous heart attack)<br>☐ Heart failure<br>☐ Peripheral vascular disease (varicose veins, vascular occlusion))<br>☐ Cardiovascular disease<br>☐ Dementia<br>☐ Chronic lung disease (asthma, COPD, tuberculosis)<br>☐ Rheumatic disease (rheumatism)<br>☐ Peptic ulcer (ulcer)<br>☐ Liver disease<br>☐ Diabetes<br>☐ Hemiplegia or paraplegia (partial or complete paralysis)<br>☐ Renal disease<br>☐ Malignancy (cancer)<br>☐ AIDS/HIV

V. QUESTIONS TO ASK TO ADOLESCENT CHILD

60. We would like you to indicate which of the situations listed at the side apply to you.<br><br>(If there is more than one option suitable for you, you can tick it.)	☐ Failure to concentrate / difficulty in focusing while listening to the teacher<br>☐ Difficulty in understanding the subjects at work and in daily life<br>☐ Angry/irritable mood<br>☐ Anxious mood<br>☐ Sad mood<br>☐ Rebellious mood<br>☐ Laziness or feeling of laziness<br>☐ Feeling of tiredness<br>☐ Indecisive mood<br>☐ Slow reaction<br>☐ Feeling like in a smokescreen<br>☐ Difficulty in communicating with others<br>☐ Headache<br>☐ Vision impairment<br>☐ Gastric complaints<br>☐ Other (please explain) ……………..…………...…………<br>☐ **NONE**	
61. Was there anything you want to do but cannot do because of your illness?	☐ Yes, there was (*Go to question 62.*)<br>☐ No there wasn't (*Go to question 63.*)	
62. Can you write down the things you want to do but cannot do due to your illness?	1) ……………………………………………………………<br>2) ……………………………………………………………<br>3) ……………………………………………………………<br>4) ……………………………………………………………<br>5) ……………………………………………………………	
63. Is your illness a barrier for your social life?	☐ Yes	☐ No
64. How often do you get angry?	☐ Very often<br>☐ Often	☐ Rarely<br>☐ Never
65. Have you ever been subjected to any bad events due to your inability to control your anger?	☐ Yes	☐ No
66. How often do you feel unhappy?	☐ Very often<br>☐ Often	☐ Rarely<br>☐ Never
67. Do you experience forgetfulness?	☐ Yes (*Go to question 68.*)<br>☐ No (*Go to question 69.*)	
68. How often do you experience forgetfulness?	☐ Very often<br>☐ Often	☐ Rarely
69. Do you encounter difficulties in your education life?	☐ Yes (*Go to question 70.*)<br>☐ No (*Go to question 71.*)	

70. Can you write the difficulties you encounter in your education life??	1) ………………………………………………………………<br>2) ………………………………………………………………<br>3) ………………………………………………………………<br>4) ………………………………………………………………<br>5) ………………………………………………………………	
71. Do you have difficulty planning?	☐ Yes	☐ No
72. Do you have difficulty making decisions when you have to make a decision about something?	☐ Yes, I do	☐ No, I don’t
