# Supplementary material for: Phenylketonuria from the perspectives of patients in Türkiye
Source: Orphanet J Rare Dis. 2024 Feb 20;19:78. doi: 10.1186/s13023-024-03079-z (PMC10880278; doi:10.1186/s13023-024-03079-z)
Supplement: Supplementary file 3 — Additional file 3: The adult data collection form in Turkish and English. [file 13023_2024_3079_MOESM3_ESM.pdf]

Fenilketonüri (PKU) Hasta Yolculuğu Araştırması

Veri Toplama Formu

Yetişkin (20 Yaş ve üzeri)

ANKETÖR (isminizin yanına çarpı işareti koyunuz)

S.D. ☐

G.Ç. ☐

Hasta Ad/Soyadı Baş Harfleri **Ebeveynin Adı/Soyadı Baş Harfleri**

....

Anket doldurma Tarihi ve Saat

....

AÇIKLAMALAR

Bu soru formunda Klasik Fenilketonüri tanısı almış ve araştırmaya katılan kişi "***Gönüllü***", gönüllü adına bu soru formuna yanıt veren ebeveyni ya da bir başkası ise "***Yakını***" olarak tanımlanmaktadır.

GÖNÜLLÜ SEÇİM ÖLÇÜTLERİ

ÇALIŞMAYA DAHİL ETME ÖLÇÜTLERİ

	Evet	Hayır
1. Klasik Fenilketonüri tanısı almış yetişkin (20 yaş üstü) gönüllüler	☐	☐
2. Çalışma hakkında bilgilendirilen ve yazılı olur veren gönüllü ya da yakını	☐	☐
3. Araştırma kapsamında kullanılacak veri toplama formunda bulunan sorulara ve değerlendirmelere yanıt vermek için entelektüel kapasiteye sahip olan gönüllü ya da yakını	☐	☐

Gönüllünün çalışmaya alınabilmesi için yukarıdaki soruların tümünün cevabı **E V E T** olmalıdır!

ÇALIŞMA DIŞI BIRAKMA ÖLÇÜTLERİ

	Evet	Hayır
1. Çalışmaya katılmayı istemeyenler.	☐	☐

Gönüllünün çalışmaya alınabilmesi için yukarıdaki soruların tümünün cevabı **H A Y I R** olmalıdır!

I. GÖNÜLLÜYE AİT TANIMLAYICI BİLGİLER

1. Doğum Tarihi	.... / .... / ....<br>Gün Ay Yıl	
2. Cinsiyeti	☐ Erkek	☐ Kadın
3. Medeni Durumu	☐ Evli	☐ Bekar
4. Sahip olduğu çocuk sayısı	………………………………………………………………	
5. Fenilketonüri tanısı alan çocuğu var mı?	☐ Evet	☐ Hayır
6. Yaşadığı İl	………………………………………………………………	
7. Son Mezun Olduğu Okul	☐ İlkokul<br>☐ Ortaokul<br>☐ Lise	☐ Üniversite<br>☐ Yüksek lisans<br>☐ Doktora
8. Mesleği	☐ Ev Hanımı<br>☐ Diğer (açıklayınız):…………………………………………………	
9. Gönüllü sağlığı ile evde yaşayanlardan kim ilgileniyor?	☐ Eşi<br>☐ Kardeşi<br>☐ Anne<br>☐ Baba<br>☐ Diğer (açıklayınız): ………………………………………………	

10. Hane halkının aylık ortalama geliri	☐ Asgari Ücret seviyesi (2.324 TL)<br>☐ 2.500 - 5.000 TL arası<br>☐ 5.001 - 10.000 TL arası<br>☐ 10.001 – 15.000 TL arası<br>☐ 15.001 TL üstü

**II. METABOLİZMA MERKEZİNE AİT BİLGİLER**	
11. Sürekli olarak kontrole gittiğiniz Metabolizma Merkezinin adı nedir?	☐ İstanbul Üniversitesi Tıp Fakültesi - İstanbul<br>☐ Cerrahpaşa Tıp Fakultesi - İstanbul<br>☐ Okmeydanı Eğitim ve Araştırma Hastanesi - İstanbul<br>☐ Marmara Üniversitesi Tıp Fakültesi - İstanbul<br>☐ Hacettepe Üniversitesi Tıp Fakültesi - Ankara<br>☐ Ankara Üniversitesi Tıp Fakültesi - Ankara<br>☐.Gazi Üniversitesi Tıp Fakültesi - Ankara<br>☐.Başkent Üniversitesi Çocuk Hastanesi - Ankara<br>☐.Ankara Şehir Hastanesi - Ankara<br>☐ Dokuz Eylül Üniversitesi Tıp Fakültesi - İzmir<br>☐ Ege Üniversitesi Tıp Fakültesi - İzmir<br>☐ Akdeniz Üniversitesi Tıp Fakültesi - Antalya<br>☐ Antalya Eğitim ve Araştırma Hastanesi - Antalya<br>☐ Çukurova Üniversitesi Tıp Fakültesi - Adana<br>☐ Adana Şehir Hastanesi - Adana<br>☐ Uludağ Üniversitesi Tıp Fakültesi - Bursa<br>☐ Van Eğitim Araştırma hastanesi - Van<br>☐ Diğer ……………………………….……<br><br>☐ **HİÇ BİR** Metabolizma Merkezine gitmiyoruz<br><br>(*12. soruya geçiniz*)
12. "**Son iki yılda**" kontrol için kaç kez Metabolizma Merkezine gittiniz?	☐ 1 ☐ 2<br>☐ 3 ☐ 4<br>☐ Daha fazla (belirtiniz): ……….
13. "**Son bir yılda**" kontrol için kaç kez Metabolizma Merkezine gittiniz?	☐ 1 ☐ 2<br>☐ 3 ☐ 4<br>☐ Daha fazla (*belirtiniz*): ……….……….….………...
14. Halen Ergenlik çağında kontrole gittiğiniz Metabolizma Merkezine mi gidiyorsunuz?	☐ **EVET**, hâlâ aynı Metabolizma Merkezine kontrole gidiyoruz *(15. soruya geçiniz)*<br>☐ **HAYIR** farklı bir Metabolizma Merkezine kontrole gidiyoruz *(16. soruya geçiniz)*<br>☐ **HİÇ BİR** Metabolizma Merkezine kontrole götürmüyoruz *(17. soruya geçiniz)*

15. Aynı Metabolizma Merkezine ne sıklıkla gidiyorsunuz?	☐ Son üç yıldır **her yıl İKİ KEREDEN FAZLA** sayıda kontrole gidiyoruz<br>☐ Son üç yıldır **her yıl EN AZ BİR KERE** kontrole gidiyoruz<br>☐ Diğer (*açıklayınız*): …………..…………..………….. (*18. soruya geçiniz.*)
16. Farklı bir Metabolizma Merkezine ne sıklıla gidiyorsunuz?	☐ Son üç yıldır **her yıl İKİ KEREDEN FAZLA** sayıda kontrole gidiyoruz<br>☐ Son üç yıldır **her yıl EN AZ BİR KERE** kontrole gidiyoruz<br>☐ Diğer (*açıklayınız*): …………..…………..………….. (*18. soruya geçiniz.*)
17. Metabolizma Merkezine neden gitmiyorsunuz?	☐ İhtiyaç duymadık<br>☐ Gitmemiz gerektiğini kimse söylemedi<br>☐ İmkanımız yok<br>☐ Diğer (*açıklayınız*): …………..…………..…………..
18. İmkanınız olsa kontrole gittiğiniz Metabolizma Merkezi ya da Metabolizma Uzmanını değiştirmek ister miydiniz?	☐ Evet, değiştirme isterdim. (1*9. soruya geçiniz*)
	☐ Hayır, değiştirmek istemezdim. (*20. soruya geçiniz*)
19. Nedenini belirtiniz	…….……………………..………………..…………….………
20. Düzenli olarak **<u>diyetisyen kontrolüne</u>** gidiyor musunuz?	☐ Evet düzenli olarak gidiyoruz (*21. soruya geçiniz*)
	☐ Hayır düzenli olarak gitmiyoruz (*22. soruya geçiniz*)
	☐ Hiçbir diyetisyene kontrole gitmiyoruz(*22. soruya geçiniz*)
21. Diyetisyene ne sıklıkla kontrole gidiyorsunuz?	☐ **Her yıl İKİ KEREDEN FAZLA** sayıda kontrole gidiyoruz.<br>☐ **Her yıl EN AZ BİR KERE** kontrole gidiyoruz<br>☐Diğer (*açıklayınız*): …………..…………..…………..
22. Diyetisyene neden gitmiyorsunuz?	☐ İhtiyaç duymadık<br>☐ Gitmemiz gerektiğini kimse söylemedi<br>☐ Diyeti kendi isteğimizle bıraktık<br>☐ Diyeti bırakmamız söylendi<br>☐ Tedavi bırakıldı<br>☐ İmkanımız yok<br>☐ Gerekli olmadığını düşünüyoruz<br>☐ Diğer (*açıklayınız*): …………..…………..…………..
23. Ergenlik döneminde hiç **<u>Psikoloğa</u>** gittiniz mi?	☐ Evet (*24. soruya geçiniz*)
	☐ Hayır (*27. soruya geçiniz*)

24. Ergenlik döneminde gittiğiniz Psikoloğa hala gidiyor musunuz?	☐ Evet aynı Psikoloğa gidiyoruz (2*5. soruya geçiniz*)<br>☐ Hayır farklı bir Psikoloğa gidiyoruz (*26. soruya geçiniz*)<br>☐….. kere gittik, ama artık gitmiyoruz. (*27. soruya geçiniz*)
25. Aynı Psikoloğa ne sıklıkla gidiyorsunuz?	☐ **Her yıl İKİ KEREDEN FAZLA**<br>☐ **Her yıl EN AZ BİR KERE**<br>☐ Diğer (*açıklayınız*): ………..………..…………..
26. Farklı Psikoloğa ne sıklıkla gidiyorsunuz?	☐ **Her yıl İKİ KEREDEN FAZLA**<br>☐ **Her yıl EN AZ BİR KERE**<br>☐ Diğer (*açıklayınız*): ………..………..…………..
27. Psikoloğa neden gitmiyorsunuz?	☐ İhtiyaç duymadık<br>☐ Gitmemiz gerektiğini kimse söylemedi<br>☐ İmkanımız yok.<br>☐ Gerekli olmadığını düşünüyoruz<br>☐ Diğer (*açıklayınız*): ………..………..…………..
28. Şu anda hizmet aldığınız Metabolizma Merkezinden memnuniyet durumunuz nedir?	☐ Çok memnunum<br>☐ Memnunum<br>☐ Memnun değiliz (*Nedenini belirtiniz*):<br>…………..…………..…..………..…………..…..………<br>…………..…………..…..………..…………..…..………
29. Şu anda hizmet aldığınız Metabolizma Uzmanından memnuniyet durumunuz nedir?	☐ Çok memnunum<br>☐ Memnunum<br>☐ Memnun değiliz (*Nedenini belirtiniz*):<br>…………..…………..…..………..…………..…..………<br>…………..…………..…..………..…………..…..………
30. Metabolizma Merkezinde aldığınız hizmetler nelerdir?	☐ Fenilalanin kan seviyesi ölçümü<br>☐ Diğer laboratuvar testleri<br>☐ Tıbbi tedavi doz ayarlaması<br>☐ Düşük proteinli Beslenme ve diyet hesaplaması önerileri<br>☐ Psikolog desteği<br>☐ Diğer (*açıklayınız*): ………..………..…………..
31. Eğer veriliyor olsaydı Metabolizma Merkezinden almak istediğiniz diğer hizmetler neler olurdu?	Açıklayınız:<br>…………..…………..…..………..…………..…..………<br>…………..…………..…..………..…………..…..………<br>…………..…………..…..………..…………..…..………<br>…………..…………..…..………..…………..…..………
32. Fenilanin kontrolü için kullandığınız seçenekler hangileridir?<br><br>(*sizin için uygun birden fazla seçenek var ise işaretleyebilirsiniz.*)	☐ Fenilalanin kısıtlı diyet<br>☐ Tıbbi amaçlı diyet ürünleri<br>☐ Büyük Nötral Aminoasit (LNAA)<br>☐ Sapropterin<br>☐ Diğer (*açıklayınız*): ………..………..…………...

33. Fenilalanin kısıtlı diyet uygulamasında karşılaştığınız zorluklar nelerdir?<br><br>(*sizin için uygun birden fazla seçenek var ise işaretleyebilirsiniz.*)	☐ Seçeneklerin az olması<br>☐ Hazırlamanın zor olması / tarif bilgilerinin olmaması<br>☐ Fenilalanin seviyesini düşürmede etkisinin az/kısıtlı olması<br>☐ Beslenme şekli sebebiyle çevreden tepki alması<br>☐ Beslenme şeklinin iş yerinde zorluklara sebep olması<br>☐ Tad ve lezzet eksikliği<br>☐ Ürünlerin pahalı olması nedeniyle erişilememesi<br>☐ Çalışma hayatı, eğitim, seyahat gibi nedenlerle diyete uyumun sosyal zorlukları (*Yaşadığınız sosyal zorluğu belirtiniz*)<br>……………………………………………………………<br>……………………………………………………………<br>……………………………………………………………<br>……………………………………………………………<br>☐ Diğer (*açıklayınız*):<br>……………………………………………………………<br>……………………………………………………………<br>……………………………………………………………<br>……………………………………………………………<br>☐ Hayır, yok
34. Düşük Proteinli ürünlerinin kullanımında karşılaştığınız zorluklar nelerdir?<br><br>(*sizin için uygun birden fazla seçenek var ise işaretleyebilirsiniz.*)	☐ Seçeneklerin az olması<br>☐ Fenilalanin seviyesini düşürmede etkisinin az/kısıtlı olması<br>☐ Tad ve lezzet eksikliği<br>☐ Pahalı olması<br>☐ Çalışma hayatı, eğitim, seyahat gibi nedenlerle diyete uyumun sosyal zorlukları (*Yaşadığınız sosyal zorluğu belirtiniz*)<br>……………………………………………………………<br>……………………………………………………………<br>……………………………………………………………<br>……………………………………………………………<br>☐ Diğer (*açıklayınız*): …………………………………………
35. Amino Asit veya LNAA kullanımında karşılaştığınız zorluklar nelerdir?<br><br>(*sizin için uygun birden fazla seçenek var ise işaretleyebilirsiniz.*)	☐ Tatlarının kötü olması<br>☐ Fenilalanin seviyesini düşürmede etkisinin az/kısıtlı olması<br>☐ Tablet sayısının fazla olması nedeniyle kullanım güçlüğü<br>☐ Diğer (*açıklayınız*): …………………………………………
36. Size, Sapropterin duyarlılık testi yapıldı mı?	☐ Hayır (*37. soruya geçiniz*)<br>☐ Evet (*38. soruya geçiniz*)
37. Yapılmamasının nedenini belirtiniz	……………………………………………………………<br>……………………………………………………………<br><br>(*40. soruya geçiniz.*)

38. Sapropterin duyarlılık testi pozitif çıktıysa size bu ilaç reçete edilerek kullanmanız sağlandı mı?	☐ Evet (*39. soruya geçiniz.*)	
	☐ Hayır<br>Nedenini belirtiniz: …………………..…..…………..…..…..<br>…..…………..………...…………..…..…………..…..…..<br>(*40. soruya geçiniz.*)	
39. Sapropterin tedavisi uygularken karşılaştığınız zorluklar var mı, varsa nelerdir?<br><br>(*sizin için uygun birden fazla seçenek var ise işaretleyebilirsiniz*)	☐ Gönüllünün Sapropterin duyarlı olmaması<br>☐ Fenilalanin seviyesini düşürmede etkisinin az/kısıtlı olması<br>☐ Yan etkileri<br>☐ Diğer (*açıklayınız*): ………..………….……….…..….<br>☐ YOK	
40. Kontrol için farklı bölüm uzmanlarına gidiyor musunuz?	☐ Evet (4*1. soruya geçiniz*)	
	☐ Hayır (*42. soruya geçiniz*)	
41. Kontrol için hangi uzmanlara, yılda kaç kez gidiyorsunuz?	☐ Psikiyatrist: ………………….kez/yıl<br>☐ Nörolog: ……………………kez/yıl<br>☐ Diğer (*açıklayınız*): ………..………….……….…..….	
42. Metabolizma merkezine erişimde zorluk yaşıyor musunuz?	☐ Evet (*43. soruya geçiniz*)	
	☐ Hayır (*44. soruya geçiniz*)	
43. Metabolizma merkezine erişimde ne tür zorluklar yaşıyorsunuz?<br><br>(*sizin için uygun birden fazla seçenek var ise işaretleyebilirsiniz.*)	☐ Metabolizma merkezinin başka şehirde olması<br>☐ Merkezin başka şehirde olması nedeniyle test sonuçları ve tedavi doz ayarlaması için ertesi gün konaklama yapma zorunluluğu olması<br>☐ Randevu almanın zorlukları olması<br>☐ Muayene ve kontrol için bekleme süresinin çok uzun olması<br>☐ Ekonomik zorluklar olması<br>☐ Diğer (*açıklayınız*): ………..………….……….…..….	
44. Kan fenilalanin değeri kontrolünü hangi kurum aracılığı ile sağlıyorsunuz?	☐ Metabolizma merkezi<br>☐ Devlet hastanesi<br>☐ Özel hastane<br>☐ Özel laboratuvar<br>☐ Diğer (*açıklayınız*): ………..………….……….…..….	
45. Kan fenilalanin değerini ne sıklıkla takip ediyorsunuz?	☐ Yılda bir<br>☐ 6 ayda bir<br>☐ 3 ayda bir	☐ Ayda bir<br>☐ Ayda iki<br>☐ Diğer (*açıklayınız*):<br>………..…………………
46. Doktor ya da diyetisyenin sizin için belirlediği hedef fenilalanin düzeyi nedir?	☐ 240 µmol/L<br>☐ 360 µmol/L<br>☐ 480 µmol/L	☐ 600 µmol/L<br>☐ Diğer (*açıklayınız*):<br>………..…………………
47. Kan fenilalanin ölçümünüz en son kaç ay önce yapıldı?	☐ 1 ay önce<br>☐ 2 ay önce	☐ 3 ay önce<br>☐ Diğer (*açıklayınız*):<br>………..…………………
48. En son ölçülen kan fenilalanin değeriniz nedir?	Belirtiniz: ……… µmol/L	

49. Daha çok bilgi sahibi olmak ya da bilgilendirilmek istediğiniz konular nelerdir?<br><br>(*sizin için uygun birden fazla seçenek var ise işaretleyebilirsiniz.*)	☐ Hastalık farkındalığı kampanyaları<br>☐ Hasta dernekleri<br>☐ Yeni tedaviler<br>☐ Yeni beslenme olanakları (yemek tarifleri gibi)<br>☐ Gıda içerikleri<br>☐ Hasta ve yakınlarına ait grup aktiviteleri<br>☐ Doktor ile daha sık görüşmek<br>☐ Diğer (*açıklayınız*): …………..…………..…………..….
50. Gönüllünün sahip olmak istediği **DİĞER İMKANLAR** nelerdir?<br><br>(*sizin için uygun birden fazla seçenek var ise işaretleyebilirsiniz.*)	☐ Hasta ailelerine PKU yönetimi konusunda eğitim olanaklarının sağlanması<br>☐ Erişim kolaylığı için daha fazla Metabolizma Merkezinin faaliyete geçmesi<br>☐ Metabolizma merkezine fenilalanin ölçümü için kan gönderme imkanı olması<br>☐ Hastaneye gitmeden telefon ve internet ile takip ve kontrol sistemi kurulması (evde kan alma, uzaktan fenilalanin ve uzman kontrolü)<br>☐ Hasta Derneğinin daha aktif olması<br>☐ Her ilde farkındalık faaliyetlerinin yapılması<br>☐ Fenilalanin seviyesinin ölçülmesi için Evde ölçüm cihazlarının bulunması<br>☐ Daha fazla ilaç tedavisi seçeneği olması<br>☐ Daha fazla düşük proteinli gıda seçeneği olması<br>☐ Toplumda fenilketonüri konusunda farkındalık yaratılması<br>☐ TV de, dizilerde PKU, yenidoğan taraması anlatılması.<br>☐ Diğer (*açıklayınız*): …………..…………..…………..….

III. SOSYAL YAŞAMA AİT ÖZELLİKLER

51. Sizin **MEVCUT DURUMDA** yaşadığınız olumlu / olumsuz bulgular var mı? Var ise nelerdir?<br><br>(sizin için uygun birden fazla seçenek var ise işaretleyebilirsiniz.)	☐ Dikkat toplayamama / odaklanma güçlüğü<br>☐ İş ve gündelik hayatta konuları anlamada güçlük çekme<br>☐ Öfkeli / sinirli ruh hali<br>☐ Endişeli ruh hali<br>☐ Üzüntülü ruh hali<br>☐ İsyankar ruh hali<br>☐ Tembellik ya da tembellik hissi<br>☐ Yorgunluk hissi<br>☐ Kararsız ruh hali<br>☐ Reaksiyon yavaşlığı<br>☐ Sis perdesi içinde olduğunu hissetmek<br>☐ Başkaları ile İletişim güçlüğü<br>☐ Baş ağrısı<br>☐ Görme bozukluğu<br>☐ Mide şikayetleri<br>☐ Diğer (açıklayınız) ....<br>☐ **YOK**	
52. Hastalığınız sebebiyle yapmak isteyip yapamadığınız bir şey oldu mu?	☐ Evet oldu (*53. soruya geçiniz.*)<br>☐ Hayır olmadı (*54. soruya geçiniz.*)	
53. Yapmak isteyip yapamadığınız şeyi veya şeyleri yazınız.	1) ....<br>2) ....<br>3) ....<br>4) ....<br>5)	
54. Hastalığınız sosyal yaşantınız için bir engel mi?	☐ Evet	☐ Hayır
55. Ne kadar sıklıkla öfkeleniyorsunuz?	☐ Çok sık<br>☐ Sık	☐ Nadiren<br>☐ Hiç
56. Öfkenizi kontrol edememeniz nedeniyle herhangi bir kötü olaya maruz kaldınız mı?	☐ Evet	☐ Hayır
57. Ne kadar sıklıkla kendinizi mutsuz hissediyorsunuz?	☐ Çok sık<br>☐ Sık	☐ Nadiren<br>☐ Hiç
58. Unutkanlık yaşıyor musunuz?	☐ Evet (*59. soruya geçiniz.*)<br>☐ Hayır (*60. soruya geçiniz.*)	
59. Ne sıklıkla unutkanlık yaşıyorsunuz?	☐ Çok sık<br>☐ Sık	☐ Nadiren
60. İş hayatınızda zorluklarla karşılaşıyor musunuz?	☐ Evet (*61. soruya geçiniz.*)<br>☐ Hayır (*62. soruya geçiniz.*)	

61. Karşılaştığınız zorlukları yazınız.	……………………………………………………………… ……………………………………………………………… ………………………………………………………………	
62. Planlama yaparken zorluk yaşıyor musunuz?	☐ Evet	☐ Hayır
63. Herhangi bir konuda karar vermeniz gerektiğinde karar verirken güçlük yaşıyor musunuz?	☐ Evet yaşıyorum	☐ Hayır yaşamıyorum

**IV. EŞLİK EDEN KRONİK HASTALIK BİLGİLERİ**	
64. Sizde bulunan ve yan tarafta listelenen hastalık ya da hastalıkları işaretleyiniz.<br><br>Sizde birden fazla hastalığın olması halinde ilgili her hastalığın yanına işaret koyunuz.	☐ Miyokart enfarktüsü (geçirilmiş kalp krizi)<br>☐ Kalp yetmezliği<br>☐ Periferik damar hastalığı (varis, damar tıkanıklığı)<br>☐ Kalp-damar hastalığı<br>☐ Demans (bunama)<br>☐ Kronik akciğer hastalığı (astım, KOAH, tüberküloz)<br>☐ Romatizmal hastalık (romatizma)<br>☐ Peptik ülser (ülser)<br>☐ Karaciğer hastalığı<br>☐ Diyabet (şeker hastalığı)<br>☐ Hemipleji ya da parapleji (kısmı ya da tam felç)<br>☐ Böbrek hastalığı<br>☐ Malignite (kanser hastalığı)<br>☐ AIDS/HIV

V. GÖNÜLLÜNÜN PARTNERİNE (EŞİ YA DA YAKININA) SORULACAK SORULAR		
65. Gönüllünün MEVCUT DURUMDA yaşadığı ve yan tarafta listelenen durumları belirtmesini istiyoruz.<br><br>(Gönülü için uygun birden fazla seçenek var ise işaretleyebilirsiniz.)	☐ Dikkat toplayamama / odaklanma güçlüğü<br>☐ İş ve gündelik hayatta konuları anlamada güçlük çekme<br>☐ Öfkeli / sinirli ruh hali<br>☐ Endişeli ruh hali<br>☐ Üzüntülü ruh hali<br>☐ İsyankar ruh hali<br>☐ Tembellik Tembellik ya da tembellik hissi<br>☐ Yorgunluk hissi<br>☐ Kararsız ruh hali<br>☐ Reaksiyon yavaşlığı<br>☐ Sis perdesi içinde olduğunu hissetmek<br>☐ Başkaları ile iletişim güçlüğü<br>☐ Baş ağrısı<br>☐ Görme bozukluğu<br>☐ Mide şikayetleri<br>☐ Diğer (*açıklayınız*): ………….…………..…………<br>☐ **YOK**	
66. Gönüllünün hastalığı sebebiyle yapmak isteyip yapamadığı bir şey oldu mu?	☐ Evet oldu (*67. soruya geçiniz.*)<br>☐ Hayır olmadı (*68. soruya geçiniz.*)	
67. Yapmak isteyip yapamadığınız şeyi veya şeyleri yazınız.	1) ……………………………………………………………<br>2) ……………………………………………………………<br>3) ……………………………………………………………<br>4) ……………………………………………………………<br>5) ……………………………………………………………	
68. Gönüllünün hastalığı sosyal yaşantısı için bir engel mi?	☐ Evet	☐ Hayır
69. Gönüllü ne kadar sıklıkla öfkeleniyor?	☐ Çok sık<br>☐ Sık	☐ Nadiren<br>☐ Hiç
70. Gönüllü öfkesini kontrol edememesi nedeniyle herhangi bir kötü olaya maruz kaldı mı?	☐ Evet	☐ Hayır
71. Gönüllü ne kadar sıklıkla kendisini mutsuz hissediyor?	☐ Çok sık<br>☐ Sık	☐ Nadiren<br>☐ Hiç
72. Gönüllü unutkanlık yaşıyor mu?	☐ Evet (*73. soruya geçiniz.*)<br>☐ Hayır (*74. soruya geçiniz.*)	
73. Gönüllü ne sıklıkla unutkanlık yaşıyor?	☐ Çok sık<br>☐ Sık	☐ Nadiren
74. Gönüllü iş hayatında zorluklarla karşılaşıyor mu?	☐ Evet (*75. soruya geçiniz.*)<br>☐ Hayır (*76. soruya geçiniz.*)	
75. Gönüllünün iş hayatında karşılaştığı zorlukları yazınız.	………………………………………………………………<br>………………………………………………………………<br>………………………………………………………………	

76. Gönüllü planlama yaparken zorluk yaşıyor mu?	☐ Evet	☐ Hayır
77. Gönüllü herhangi bir konuda karar vermesi gerektiğinde karar verirken güçlük yaşıyor mu?	☐ Evet yaşıyor	☐ Hayır yaşamıyor

Phenylketonuria: The Patient’s Journey Study

Data Collection Form

Adult Group (20 Years of Age and Over)

INTERVIEWER (Put a cross next to your name)

S.D. ☐

G.Ç. ☐

Initials of the Patient’s Name/Surname **Initials of the Parent's Name/Surname**

....

Survey Completion Date and Time

....

EXPLANATIONS

In this questionnaire, the person diagnosed with Classical Phenylketonuria and participating in the research is defined as "***Volunteer*****",** and the parent or someone else who answers this questionnaire on behalf of the volunteer is defined as "***Relative***".

VOLUNTEER SELECTION CRITERIA

STUDY INCLUSION CRITERIA

	Yes	No
1. Adult volunteers (over 20 years old) diagnosed with Classical Phenylketonuria	☐	☐
2. The volunteer or his/her relative who was informed about the study and gave written consent	☐	☐
3. The volunteer or his/her relative who has the intellectual capacity to answer the questions and evaluations in the data collection form to be used within the scope of the study	☐	☐

In order for the volunteer to be included in the study, the answer to all of the above questions must be **Y E S!**

STUDY EXCLUSION CRITERIA

	Yes	No
1. Those who do not want to participate in the study.	☐	☐

In order for the volunteer to be included in the study, the answer to all of the above questions must be **N O!**!

I. DESCRIPTIVE INFORMATION ABOUT THE VOLUNTEER

1. Date of Birth	.... / .... / ....<br>Day Month Year	
2. Gender	☐ Male	☐ Female
3. Marital Status	☐ Married	☐ Single
4. Number of children he/she has	……………………………………………………………	
5. Does she/he have a child diagnosed with phenylketonuria?	☐ Yes	☐ No
6. City of residence	……………………………………………………………	
7. The last school he/she graduated from	☐ Primary school<br>☐ Secondary school<br>☐ High school	☐ University<br>☐ Post graduate<br>☐ Doctorate
8. Occupation	☐ Housewife<br>☐ Other (please explain):…………………………………………	
9. Among the people living in the house, who takes care about the volunteer's health?	☐ Spouse<br>☐ Sibling<br>☐ Mother<br>☐ Father<br>☐ Other (please explain): ……………………………………	

10. Average monthly income of the household	☐ Minimum Wage level (2.324 TL)<br>☐ Between 2.500 - 5.000 TL<br>☐ Between 5.001 - 10.000 TL<br>☐ Between 10.001 – 15.000 TL<br>☐ Over 15.001 TL

**II. INFORMATION ABOUT THE METABOLISM CENTER**	
11. What is the name of the Metabolism Center where you constantly visit for control?	☐ İstanbul University School of Medicine - İstanbul<br>☐ Cerrahpaşa School of Medicine - İstanbul<br>☐ Okmeydanı Training and Research Hospital - İstanbul<br>☐ Marmara University School of Medicine - İstanbul<br>☐ Hacettepe University School of Medicine - Ankara<br>☐ Ankara University School of Medicine - Ankara<br>☐.Gazi University School of Medicine - Ankara<br>☐.Başkent University Children's Hospital - Ankara<br>☐.Ankara City Hospital - Ankara<br>☐ Dokuz Eylül University School of Medicine - İzmir<br>☐ Ege University School of Medicine - İzmir<br>☐ Akdeniz University School of Medicine - Antalya<br>☐ Antalya Training and Research Hospital - Antalya<br>☐ Çukurova University School of Medicine - Adana<br>☐ Adana City Hospital - Adana<br>☐ Uludağ University School of Medicine - Bursa<br>☐ Van Training and Research Hospital - Van<br>☐ Other ……….………………….……<br><br>☐ **Neither of them** We do not go to a Metabolism Center for control.<br>(*Go to question 12)*
12. How many times have you visited the Metabolism Center for control in the "**last two years**"?	☐ 1 ☐ 2<br>☐ 3 ☐ 4<br>☐ More (specify) ……….
13. "How many times have you visited the Metabolism Center for control in the "**last year"**?	☐ 1 ☐ 2<br>☐ 3 ☐ 4<br>☐ More (specify) …….….……….…………..
14. Do you still visit the Metabolism Center where you went for control during adolescence?	☐ **YES**, we still visit the same Metabolism Center for control. *(Go to question 15)*<br>☐ **NO,** we are visiting a different Metabolism Center for control. *(Go to question 16)*<br>☐ **Neither of them** We do not visit a Metabolism Center for control (*Go to question 17)*

15. How often do you visit the same Metabolism Center?	☐ We have been going for control **MORE THAN TWICE every year** for the last three years.<br>☐ We have been going for control **AT LEAST ONCE a year** for the last three years.<br>☐ Other (please explain): ……………………………….<br>*(Go to question 18)*
16. How often do you go to another Metabolism Center?	☐ We have been going for control **MORE THAN TWICE every year** for the last three years<br>☐ We have been going for control **AT LEAST ONCE a year** for the last three years.<br>☐ Other (please explain) …………...…………...…………...<br>(*Go to question 18*)
17. Why don't you go to a Metabolism Center?	☐ We didn't need to.<br>☐ Nobody told us that we should go<br>☐ We don't have the financial means<br>☐ Other (please explain) …………...…………...…………..…
18. If you had the opportunity, would you want to change the Metabolism Center or Metabolism Specialist you visit for control?	☐ Yes, I would like to change.<br>(*Go to question 19*)
	☐ No, I wouldn't want to change.<br>(*Go to question 20*)
19. Please state the reason.	……..………………..……………..………………..……
20. Do you **<u>visit a dietitian</u>** regularly?	☐ Yes, we go regularly (*Go to question 21*)
	☐ No, we don't go regularly (*Go to question 22*)
	☐ We do not visit any dietician for control. (*Go to question 22*)
21. How often do you visit a dietitian for control?	☐ We are going for control **MORE THAN TWICE every year**<br>☐ We are going for control **AT LEAST ONCE a year**<br>☐ Other (please explain) …………...…………………..
22. Why don't you visit a dietician?	☐ We didn't need to<br>☐ Nobody told us that we should go<br>☐ We left the diet voluntarily.<br>☐ We were told to stop dieting<br>☐ Treatment was stopped<br>☐ We don't have the financial means<br>☐ We think it is not necessary<br>☐ Other (please explain) …………...……………...…………
23. Have you ever visited a **<u>Psychologist</u>** during	☐ Yes (*Go to question 24*)

adolescence?	☐ No (*Go to question 27*)
24. Do you still go to the psychologist you visited during adolescence?	☐ Yes, we go to the same Psychologist (go to question 25)<br>☐ No, we are going to a different Psychologist (Go to question 26)<br>We went ….times, but we don't go anymore. (Go to question 27)
25. How often do you visit the same psychologist??	☐ **MORE THAN TWICE every year**<br>☐ **AT LEAST ONCE a year**<br>☐ Other (please explain) …………...…………..…………..
26. How often do you visit a different psychologist?	☐ **MORE THAN TWICE every year**<br>☐ **AT LEAST ONCE a year**<br>☐ Other (please explain) …………..…………..…………..
27. Why don't you visit a psychologist?	☐ We didn't need to<br>☐ Nobody told us that we should go<br>☐ We don't have the financial means<br>☐ We think it is not necessary<br>☐ Other (please explain) …………..…………..…………..
28. What is your satisfaction level with the Metabolism Center you are currently receiving service from?	☐ I am very satisfied<br>☐ I am satisfied<br>☐ We are not satisfied (specify why).………..………..………..…..………<br>…………..…………...…..………..…………..…
29. What is your satisfaction level with the Metabolism Specialist you are currently receiving service from?	☐ I am very satisfied<br>☐ I am satisfied<br>☐ We are not satisfied (specify why).………..………..………..…..………<br>…………..…………...…………..…..………<br>…………..…………...…..………..…………..…..………
30. What services do you receive from the Metabolism Center?	☐ Phenylalanine blood level measurement<br>☐ Other laboratory tests<br>☐ Medical treatment dose adjustment<br>☐ Recommendations for low protein diet and diet calculation<br>☐ Psychological support<br>☐ Other (please explain) …..………..…………..
31. If provided, what other services would you like to receive from the Metabolism Center?	Please explain:<br>…………..…………...…..………..…………..…..………<br>…………..…………...…..………..…………..…..………<br>…………..…………...…..………..…………..…..………<br>…………..…………...…..………..…………..…..………

32. What options do you use to control phenylalanine?<br><br>(If there is more than one option suitable for you, you can tick it.)	☐ Phenylalanine-restricted diet<br>☐ Dietary products for medical purposes<br>☐ Large Neutral Amino Acid (LNAA)<br>☐ Sapropterin<br>☐ Other (please explain) ……………………..
33. What are the difficulties you encounter in applying a phenylalanine restricted diet?<br><br>(If there is more than one option suitable for you, you can tick it.)	☐ There are few options available<br>☐ It is difficult to prepare / lack of recipe information<br>☐ It has little/limited effect on reducing phenylalanine levels<br>☐ Reaction from the environment due to diet<br>☐ Diet is causing difficulties at workplace<br>☐ Lack of taste and flavor<br>☐ Inaccessibility of products due to their expensiveness<br>☐ Social difficulties in adapting to the diet due to reasons such as working life, education, travel (Indicate the social difficulty you experience)<br>…………………………………………………………………<br>…………………………………………………………………<br>…………………………………………………………………<br>☐ Other (please explain)<br>……………………………………………………………………<br>……………………………………………………………………<br>………………………………………………………………<br>☐ No, there is none
34. What are the difficulties you encounter in the use of low-protein products?<br><br>(If there is more than one option suitable for you, you can tick it.)	☐ There are few options available<br>☐ They have little/limited effect on reducing phenylalanine levels<br>☐ Lack of taste and flavor<br>☐ Being expensive<br>☐ Social difficulties in adapting to the diet due to reasons such as working life, education, travel (Indicate the social difficulty you experience)<br>…………………………………………………………………<br>…………………………………………………………………<br>…………………………………………………………………<br>…………………………………………………………………<br>☐ Other (please explain)………..
35. What are the difficulties do you face in using Amino Acid or LNAA?<br><br>(If there is more than one option suitable for you, you can tick it.)	☐ They have bad taste<br>☐ They have little/limited effect on reducing phenylalanine levels<br>☐ Difficulty of use due to the large number of tablets<br>☐ Other (please explain)………………………………………
36. Have you undergone Sapropterin sensitivity testing?	☐ No (*Go to question 37*)<br>☐ Yes (*Go to question 38)*

37. State the reason why it was not done	……………………………………………………………………………<br>……………………………………………………………………………<br><br>(*Go to question 40).*
38. If the sapropterin sensitivity test was positive, were you prescribed this medicine and allowed to use it?	☐ Yes (*Go to question 39*)
	☐ No<br>Please state the reason<br>……………………………………………………<br>……………………………………………………………………………<br>(*Go to question 40*)
39. Are there any difficulties you encounter while applying sapropterin treatment? If so, what are they?<br><br>(If there is more than one option suitable for you, you can tick it.)	☐ Volunteer is not sensitive to Sapropterin<br>☐ It has little/limited effect on reducing phenylalanine levels<br>☐ Side effects<br>☐ Other (please explain) ……………………………………<br>☐ NONE
40. Do you visit different specialists for control?	☐ Yes (*Go to question 41*)
	☐ No (*Go to question 42*)
41. Which specialists do you visit for control and how many times a year?	☐ Psychiatrist: ………………….times/year<br>☐ Neurologist: ……………………times/year<br>☐ Other (please explain) ……………..
42. Do you have difficulty accessing the metabolism center?	☐ Yes (*Go to question 43*)
	☐ No (*Go to question* 44)
43. What difficulties do you experience in accessing the metabolism center?<br><br>(If there is more than one option suitable for you, you can tick it.)	☐ Metabolism center is located in another city<br>☐ Since the center is located in another city, it is necessary to stay the next day for test results and treatment dose adjustment.<br>☐ Having difficulties in getting an appointment<br>☐ Long waiting period for examination and control<br>☐ Economic hardship<br>☐ Other (please explain)……………………………………
44. Through which institution do you check your blood phenylalanine level?	☐ Metabolism center<br>☐ Public Hospital<br>☐ Private hospital<br>☐ Private laboratory<br>☐ Other (please explain) ……………………………………
45. How often do you go for the control of your blood phenylalanine level?	☐ Once a year<br>☐ Every 6 months<br>☐ Every 3 months<br>☐ Monthly<br>☐ Twice a month<br>☐ Other (please explain)<br>……………………………….

46. What is the target phenylalanine level determined by your doctor or dietician?	☐ 240 µmol/L<br>☐ 360 µmol/L<br>☐ 480 µmol/L	☐ 600 µmol/L<br>☐ Other (please explain): ………..…………………
47. How many months ago was your last blood phenylalanine measurement?	☐ 1 month ago<br>☐ 2 months ago	☐ 3 months ago<br>☐ Other (please explain) ……..…………………
48. What is your last blood phenylalanine level?	Please specify:: ……… µmol/L	
49. What are the topics that you feel lacking and/or desired to have more information about?<br><br>(If there is more than one option suitable for you, you can tick it.)	☐ Campaigns for disease awareness<br>☐ Patient societies<br>☐ Novel therapies<br>☐ New dietary opportunities (such as recipes)<br>☐ Food contents<br>☐ Group activities for patients and patient relatives<br>☐ More frequent contact with doctor<br>☐ Other (please explain) …………..…………..…………..….	
50. What are the **OTHER OPPORTUNITIES** that you would like to have?<br><br>(If there is more than one option suitable for you, you can tick it.)	☐ Providing the patient families with educational opportunity on PKU management<br>☐ Activation of more metabolism centers for ease of access<br>☐ Opportunity of sending blood samples to metabolism center for phenylalanine measurement<br>☐ Opportunity of control/follow-up without visiting a hospital (control via phone-call and internet /follow-up system, blood collection at home, etc.)<br>☐ More active patient society<br>☐ Awareness activities in every province<br>☐ Availability of home devices for phenylalanine measurement<br>☐ Availability of more options for drug therapy<br>☐ More options for low-protein foods<br>☐ Raising awareness about phenylketonuria in the society<br>☐ Programs about PKU and newborn screening on television/TV series.<br>☐ Other (please explain)………………………	

III. CHARACTERISTICS RELATED TO SOCIAL LIFE		
51. Are there any positive/negative symptoms you experience in the **CURRENT SITUATION**? If so, what are they?<br><br>(If there is more than one option suitable for you, you can tick it.)	☐ Failure to concentrate / difficulty in focusing<br>☐ Difficulty in understanding the subjects at work and in daily life<br>☐ Angry/irritable mood<br>☐ Anxious mood<br>☐ Sad mood<br>☐ Rebellious mood<br>☐ Laziness or feeling of laziness<br>☐ Feeling of tiredness<br>☐ Indecisive mood<br>☐ Slow reaction<br>☐ Feeling like in a smokescreen<br>☐ Difficulty in communicating with others<br>☐ Headache<br>☐ Vision impairment<br>☐ Gastric complaints<br>☐ Other (please explain) ……………..……………...…………<br>☐ **NONE**	
52. Was there anything you would like to do but couldn't do because of your illness?	☐ Yes, there was (*Go to question 53.*)<br>☐ No there wasn't (*Go to question 54.*)	
53. Write down the thing or things you want to do.	1) ……………………………………………………………<br>2) ……………………………………………………………<br>3) ……………………………………………………………<br>4) ……………………………………………………………<br>5) ……………………………………………………………	
54. Is your illness a barrier for your social life?	☐ Yes	☐ No
55. How often do you get angry?	☐ Very often<br>☐ Often	☐ Rarely<br>☐ Never
56. Have you ever been subjected to any bad events due to your inability to control your anger?	☐ Yes	☐ No
57. How often do you feel unhappy?	☐ Very often<br>☐ Often	☐ Rarely<br>☐ Never
58. Do you experience forgetfulness?	☐ Yes (*Go to question 59.*)<br>☐ No (*Go to question 60.*)	
59. How often do you experience forgetfulness?	☐ Very often<br>☐ Often	☐ Rarely
60. Do you encounter difficulties in your business life?	☐ Yes (*Go to question 61.*)<br>☐ No (*Go to question 62.*)	

61. Write down the difficulties you encountered.	………………………………………………………………… ………………………………………………………………… …………………………………………………………………	
62. Do you have difficulty planning?	☐ Yes	☐ No
63. Do you have difficulty making decisions when you have to make a decision about something?	☐ Yes, I do.	☐ No, I don't

**IV. INFORMATION ON COMORBID CHRONIC DISEASES**	
64. Please tick the disease(s) listed on the side that you have.<br><br>If you have more than one disease, put a check mark next to each disease.	☐ Myocardial infarct (previous heart attack)<br>☐ Heart failure<br>☐ Peripheral vascular disease (varicose veins, vascular occlusion))<br>☐ Cardiovascular disease<br>☐ Dementia<br>☐ Chronic lung disease (asthma, COPD, tuberculosis)<br>☐ Rheumatic disease (rheumatism)<br>☐ Peptic ulcer (ulcer)<br>☐ Liver disease<br>☐ Diabetes<br>☐ Hemiplegia or paraplegia (partial or complete paralysis)<br>☐ Renal disease<br>☐ Malignancy (cancer)<br>☐ AIDS/HIV

V. QUESTIONS TO ASK THE VOLUNTEER'S PARTNER (SPOUSE OR RELATIVE)

65. We would like you to indicate the situations the volunteer is CURRENTLY experiencing listed on the side.<br><br>(If there is more than one option suitable for the volunteer, you can tick it.)	☐ Failure to concentrate / difficulty in focusing<br>☐ Difficulty in understanding the subjects at work and in daily life<br>☐ Angry/irritable mood<br>☐ Anxious mood<br>☐ Sad mood<br>☐ Rebellious mood<br>☐ Laziness or feeling of laziness<br>☐ Feeling of tiredness<br>☐ Indecisive mood<br>☐ Slow reaction<br>☐ Feeling like in a smokescreen<br>☐ Difficulty in communicating with others<br>☐ Headache<br>☐ Vision impairment<br>☐ Gastric complaints<br>☐ Other (please explain) ……………………………………<br>☐ **NONE**	
66. Was there anything the volunteer would like to do but couldn't do because of her/his illness?	☐ Yes, there was (*Go to question 67.*)<br>☐ No there wasn't (*Go to question 68.*)	
67. Write down the thing or things the volunteer wants to do but cannot do.	1) ……………………………………………………<br>2) ……………………………………………………<br>3) ……………………………………………………<br>4) ……………………………………………………<br>5) ……………………………………………………	
68. Is the volunteer's illness a barrier for his/her social life?	☐ Yes	☐ No
69. How often does the volunteer get angry?	☐ Very often<br>☐ Often	☐ Rarely<br>☐ Never
70. Has the volunteer ever been subjected to any bad events due to his/her inability to control his/her anger?	☐ Yes	☐ No
71. How often does the volunteer feel unhappy?	☐ Very often<br>☐ Often	☐ Rarely<br>☐ Never
72. Does the volunteer experience forgetfulness?	☐ Yes (*Go to question 73.*)<br>☐ No (*Go to question 74.*)	
73. How often does the volunteer experience forgetfulness?	☐ Very often<br>☐ Often	☐ Rarely
74. Does the volunteer encounter difficulties in his/her business life?	☐ Yes (*Go to question 75.*)<br>☐ No (*Go to question 76.*)	

75. Write down the difficulties the volunteer encountered in his/her business life.	……………………………………………………………… ………………………………………………………………	
76. Does the volunteer have difficulty planning?	☐ Yes	☐ No
77. Does the volunteer have difficulty making decisions when she/he has to make a decision about something?	☐ Yes, he/she does.	☐ No, , he/she doesn't.
